# BiTE‐Secreting CAR‐*γδ*T as a Dual Targeting Strategy for the Treatment of Solid Tumors

**DOI:** 10.1002/advs.202206856

**Published:** 2023-04-20

**Authors:** Shi‐Wei Huang, Chih‐Ming Pan, Yu‐Chuan Lin, Mei‐Chih Chen, Yeh Chen, Chia‐Ing Jan, Chung‐Chun Wu, Fang‐Yu Lin, Sin‐Ting Wang, Chen‐Yu Lin, Pei‐Ying Lin, Wei‐Hsaing Huang, Yu‐Ting Chiang, Wan‐Chen Tsai, Ya‐Hsu Chiu, Ting‐Hsun Lin, Shao‐Chih Chiu, Der‐Yang Cho

**Affiliations:** ^1^ Translational Cell Therapy Center Department of Medical Research China Medical University Hospital Taichung 40447 Taiwan; ^2^ Institute of New Drug Development China Medical University Taichung 40447 Taiwan; ^3^ Department of Pathology Kaohsiung Veterans General Hospital Kaohsiung 813414 Taiwan; ^4^ Department of Dermatology Taichung Veterans General Hospital Taichung 40447 Taiwan; ^5^ Department of Gastroenterology Taichung Veterans General Hospital Taichung 40447 Taiwan; ^6^ Graduate Institute of Biomedical Sciences China Medical University Taichung 40447 Taiwan; ^7^ Department of Neurosurgery China Medical University Hospital Taichung 40447 Taiwan

**Keywords:** antigen heterogenicity, bispecific T‐cell engager, chimeric antigen receptor, gamma‐delta T, mRNA, nanobody

## Abstract

HLA‐G is considered as an immune checkpoint protein and a tumor‐associated antigen. In the previous work, it is reported that CAR‐NK targeting of HLA‐G can be used to treat certain solid tumors. However, the frequent co‐expression of PD‐L1 and HLA‐G) and up‐regulation of PD‐L1 after adoptive immunotherapy may decrease the effectiveness of HLA‐G‐CAR. Therefore, simultaneous targeting of HLA‐G and PD‐L1 by multi‐specific CAR could represent an appropriate solution. Furthermore, gamma‐delta T (*γδ*T) cells exhibit MHC‐independent cytotoxicity against tumor cells and possess allogeneic potential. The utilization of nanobodies offers flexibility for CAR engineering and the ability to recognize novel epitopes. In this study, V*δ*2 *γδ*T cells are used as effector cells and electroporated with an mRNA‐driven, nanobody‐based HLA‐G‐CAR with a secreted PD‐L1/CD3*ε* Bispecific T‐cell engager (BiTE) construct (Nb‐CAR.BiTE). Both in vivo and in vitro experiments reveal that the Nb‐CAR.BiTE‐*γδ*T cells could effectively eliminate PD‐L1 and/or HLA‐G‐positive solid tumors. The secreted PD‐L1/CD3*ε* Nb‐BiTE can not only redirect Nb‐CAR‐*γδ*T but also recruit un‐transduced bystander T cells against tumor cells expressing PD‐L1, thereby enhancing the activity of Nb‐CAR‐*γδ*T therapy. Furthermore, evidence is provided that Nb‐CAR.BiTE redirectes *γδ*T into tumor‐implanted tissues and that the secreted Nb‐BiTE is restricted to the tumor site without apparent toxicity.

## Graphical abstract

Elevated PD‐L1 in solid tumors increases the risk of immune escape from HLA‐G‐CAR cell therapy. The bicistronic mRNA construct that drives PD‐L1 Nb‐BiTE and HLA‐G Nb‐CAR in *γδ*T cells via electroporation is designed to address this issue. This Nb‐CAR.BiTE‐*γδ*T therapy can overcome HLA‐G and PD‐L1 dilemma and even kill tumor cells with inadequate antigen expression, resulting in potent anti‐tumor activity without apparent toxicity.

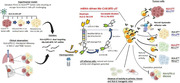

## 1 Introduction

Chimeric antigen receptor‐T (CAR‐T) cell therapy has achieved remarkable success in the clinical treatment of hematological malignancies. However, issues remain to be resolved in the use of CAR‐T for treating patients with solid tumors. The shortage of unique antigens specifically expressed in the heterogenous tumor cells may cause off‐tumor toxicity and immune escape after CAR‐T therapy.^[^ ^1^ ^]^ The immunosuppressive tumor microenvironment (TME) may obstruct CAR‐T efficacy through multiple mechanisms, such as interfering with CAR‐T trafficking to the tumor sites and increasing the expression of inhibitory receptors on effector cells to suppress the cytotoxicity of CAR‐T.^[^ ^2^ ^]^

Upregulation of immune checkpoint protein (ICP) is an immune escape mechanism by which cancer cells resist immune system attack.^[^ ^3^, ^4^ ^]^ In addition to immunotherapy employing monoclonal antibodies targeting ICPs, the development of CAR‐T therapy targeting ICP pathways has been recognized as a promising strategy for overcoming the hostile TME by converting immuno‐suppressive signals to active signals.^[^ ^5^, ^6^, ^7^ ^]^ However, the limitation of these ICP‐targeted CAR‐T therapies is related to the manifestation of ICPs in some normal tissues and the associated increases in safety concerns.^[^ ^8^ ^]^ Accordingly, an ideal target for CAR‐immune cell therapy is either a tumor‐associated antigen (TAA) or a tumor‐specific antigen (TSA) for overcoming the TME and reducing off‐tumor toxicity.

Human leukocyte antigen G (HLA‐G) is a neo‐expressed TAA in a large proportion of solid tumors, and it is also considered a potent ICP.^[^ ^9^ ^]^ In our previous study, we developed a scFv‐based HLA‐G CAR‐NK cell therapy and revealed the significant efficacy of HLA‐G CAR in the treatment of solid tumors.^[^ ^10^ ^]^ Another study also indicated that HLA‐G represents an effective target for CAR‐T therapy to eliminate tumor cells and HLA‐G‐positive suppressive cells as well.^[^ ^11^ ^]^ Although these findings have shown that HLA‐G is an effective therapeutic target and confirmed the potential of HLA‐G CAR cells in the treatment of solid tumors, we found adaptive resistance due to PD‐L1 upregulation in residual tumor cells after they were challenged by anti‐HLA‐G CAR effector cells. Our findings are in line with recent studies showing that tumor cells upregulate PD‐L1 to escape the cytotoxicity of CAR‐T therapy^[^ ^12^, ^13^ ^]^ and that tumor‐infiltrated CAR‐T cell exhaustion is associated with increasing levels of PD‐1 (PD‐L1 receptor) on effector cells and PD‐L1 in the TME.^[^ ^12^, ^13^, ^14^, ^15^, ^16^ ^]^ Consequently, the development of combined treatment strategies of PD‐L1/PD‐1 blockade and CAR‐T therapy has been reported.^[^ ^12^, ^13^, ^16^ ^]^

Bispecific T‐cell engager (BiTE) is an artificial bispecific antibody construct that simultaneously binds a surface antigen on tumor cells and a surface molecule (i.e., CD3*ε*) on T cells. Based on its advantages, such as off‐the‐shelf or on‐demand treatment, BiTE is considered highly competitive with CAR‐T therapy.^[^ ^17^ ^]^ However, tumor antigen heterogenicity and hostile TMEs are challenges for both CAR‐T and BiTE therapies.^[^ ^18^ ^]^ A recent study showed that locally secreted BiTE can complement CAR‐T cells against antigen heterogeneous solid tumors.^[^ ^19^ ^]^ BiTEs can engage tumor cells to T cells, thereby enhancing the activity of CAR‐T therapy.^[^ ^17^, ^18^, ^19^, ^20^, ^21^ ^]^ In addition, BiTEs can be designed to target ICPs to combat solid tumors.^[^ ^22^, ^23^ ^]^ Therefore, the combination of CAR‐T and BiTE targeting ICPs may overcome the dilemma of treating solid tumors.

Gamma‐delta (*γδ*) T cells can induce cytotoxicity directly through cell surface receptors, including *γδ*T‐cell receptor (TCR*γδ*) and natural killer group 2D (NKG2D), and they can also act as antigen‐presenting cells to cultivate adaptive immunity to specific antigens.^[^ ^24^, ^25^, ^26^ ^]^ Accordingly, CAR‐modified *γδ*T cells may exhibit both CAR‐directed and major histocompatibility complex (MHC)‐independent anti‐tumor activities.^[^ ^25^, ^27^, ^28^, ^29^ ^]^ In addition, the MHC‐independent property of *γδ*T cells implies that they may not cause graft‐versus‐host disease (GvHD).^[^ ^29^ ^]^ These features highlight the potential of CAR‐*γδ*T cells as the “off‐the‐shelf” products for cancer immunotherapy.

**Scheme 1:**
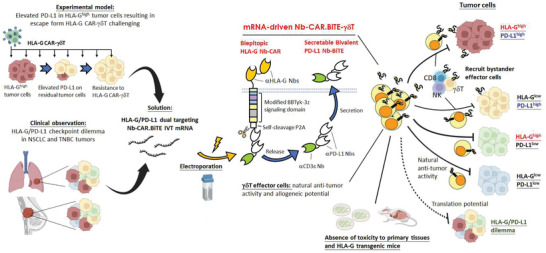
Elevated PD‐L1 in solid tumors increases the risk of immune escape from HLA‐G‐CAR cell therapy. The bicistronic mRNA construct that drives PD‐L1 Nb‐BiTE and HLA‐G Nb‐CAR in *γδ*T cells via electroporation was designed to address this issue. This Nb‐CAR.BiTE‐*γδ*T therapy can overcome HLA‐G and PD‐L1 dilemma and even kill tumor cells with inadequate antigen expression, resulting in potent anti‐tumor activity without apparent toxicity.

Since T‐cell‐secreted BiTE may not cause safety concerns,^[^ ^30^, ^31^ ^]^ targeting the PD‐L1/PD‐1 axis may avoid the exhaustion and immune escape associated with CAR‐T therapy. We incorporated a bicistronic mRNA construct encoding a nanobody (Nb)‐based HLA‐G CAR followed by a secretable Nb‐based PD‐L1‐targeting BiTE. Furthermore, V*δ*2 *γδ*T was used as an effector cell carrying the Nb‐CAR.BiTE construct to address the limitations of autologous CAR‐T and complement CAR‐T and BiTE therapies by facilitating CAR‐independent natural anti‐tumor activity when antigen expression is inadequate.^[^ ^27^ ^]^ We identified the up‐regulation of PD‐L1 in residual tumors following HLA‐G Nb‐CAR‐*γδ*T treatment in NOD/scid gamma (NSG) mice and the frequent pairing of PD‐L1 and HLA‐G expression in patient tumor lesions. The efficacy of Nb‐CAR.BiTE‐*γδ*T in the treatment of solid tumors, especially those with PD‐L1 and HLA‐G expression variants, was further evaluated. The bystander effect of secreting Nb‐BiTE on recruiting immune cells against PD‐L1‐expressing tumor cells was also assessed. Finally, the biodistribution and toxicity risks of Nb‐CAR.BiTE‐*γδ*T were also evaluated (**Scheme** **1**).

## 2 Results

### 2.1 PD‐L1 Is Upregulated in Persistent Tumor Cells after HLA‐G CAR‐*γδ*T Challenge

**Figure 1:**
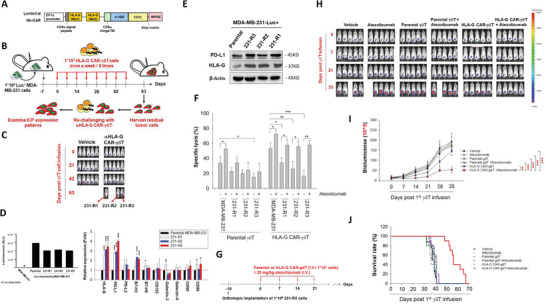
PD‐L1 blockade reinforces anti‐HLA‐G Nb‐CAR‐*γδ*T to eliminate PD‐L1‐overexpressing immune escape variants. A) Schematic diagram of anti‐HLA‐G CAR construct. HLA‐G‐targeted Nb#1 and Nb#2 were ligated with CD8*α* hinge/TM, followed by a 4‐1BB and a CD3*ξ* intracellular domains, which was driven by EF‐1*α* promoter using lentiviral vector. B) Generation of immune escape variants following anti‐HLA‐G CAR‐*γδ*T challenge in vivo. Briefly, NSG mice (*n* = 5) were orthotopically implanted with luciferase‐expressing MDA‐MB‐231 cells. After 7 d, the mice were infused with anti‐HLA‐G Nb‐CAR‐*γδ*T cells (1 × 10^7^) via tail vein injection weekly for 8 weeks. The mice were sacrificed at 63 days after the first challenge of Nb‐CAR‐*γδ*T, and then residual tumors were harvested. Subsequently, the isolated cells were re‐challenged with anti‐HLA‐G Nb‐CAR‐*γδ*T at an E:T ratio of 3:1 for 72 h. C–E) Three immune escape variants generated from individual mice showed consistently upregulated PD‐L1 levels. C) Bioluminescent signals were detected using IVIS after the MDA‐MB‐231 tumor‐bearing mice were treated with or without HLA‐G‐targeted Nb‐CAR‐*γδ*T cells. D) Bioluminescence of the residual clones (231‐R1, ‐R2, ‐R3) was measured using IVIS. Parental MDA‐MB‐231 and MDA‐MB‐231‐luc cells were used as the control (left). Expression levels of ICPs were determined through flow cytometry (right). E) PD‐L1 expression in these clones was also confirmed by immunoblotting. F) Combination with atezolizumab enhanced anti‐HLA‐G Nb‐CAR‐*γδ*T‐induced cytotoxicity against the immune escape variants. Parental MDA‐MB‐231, 231‐R1, ‐R2, and ‐R3 cells were pretreated with or without 10 µg mL^−1^ atezolizumab for 15 min and followed by coculture with or without parental or anti‐HLA‐G CAR‐*γδ*T cells for 48 h at an E:T ratio of 3:1. Subsequently, we determined specific lysis through a LIVE/DEAD cell‐mediated cytotoxicity assay. G‐I) PD‐L1 blockade reverses the sensitivity of 231‐R3 tumor cells to anti‐HLA‐G CAR‐*γδ*T challenge in vivo. G) NSG mice (*n* = 5) were orthotopically implanted with 231‐R3 cells. After 10 d, the mice were injected with or without atezolizumab (20 mg kg^−1^) combined with parental or anti‐HLA‐G CAR‐*γδ*T weekly for 4 weeks through the tail vein. H,I) Bioluminescence of the 231‐R3 tumors was measured (H,I), and J) survival rates were recorded. These results suggested that PD‐L1 is upregulated in persistent tumor cells after HLA‐G CAR‐*γδ*T challenge, and the blockade of PD‐L1 restored their sensitivity to HLA‐G CAR‐*γδ*T cells. Results are representative of at least three independent experiments. Data represent the mean ± SD, *n* = 4; *p < 0.05; **p < 0.01; ***p < 0.001; and #, not detectable based on paired Student's t‐tests. For tumor growth and survival rate comparisons, the Kaplan–Meier method and log‐rank test were performed.

Our previous study showed that targeting HLA‐G by CAR‐NK switches inhibitory signals to ablate solid tumor cells.^[^ ^10^ ^]^ Although the tumors were remarkably suppressed and the survival of the xenografted mice was significantly improved, the tumors were not eradicated, even after repeated infusions of CAR‐NK in mice. In this study, a similar outcome was observed in vivo in which MDA‐MB‐231‐Luc inoculated tumors were not completely eliminated after eight consecutive weekly injections with HLA‐G‐targeted Nb‐CAR‐*γδ*T cells (1 × 10^7^ per mice, *n* = 5) (**Figure** **1**A–C). Tumor cells isolated from each xenografted mouse were subsequently challenged with three runs of Nb‐CAR‐*γδ*T cells, which was followed by the assessment of luciferase activity (Figure 1D, left panel) to determine whether these three isolated cells were derived from the implanted luciferase‐expressing MDA‐MB‐231 cells from the mice. We hypothesized that the development of resistance to HLA‐G Nb‐CAR‐*γδ*T treatment in tumor cells may be caused by altering the expression levels of HLA‐G and/or other ICPs. Thus, we examined the expression patterns of ICPs in the cells of these three sub‐clones isolated from persistent tumors in the HLA‐G Nb‐CAR‐*γδ*T‐treated mice and compared the findings with that of the parental MDA‐MB‐231‐luc cells. The three sub‐clones maintained high levels of HLA‐G and PD‐L1, and two of these three clones (231‐R2 and ‐R3) showed increased B7‐H3. However, other ICPs, such as B7‐H6 and CD86, did not show significant increases in these sub‐clones (Figure 1D, right panel). The immunoblotting analysis also showed that PD‐L1 and HLA‐G were upregulated in all three sub‐clones (Figure 1E). We also determined whether these three isolated MDA‐MB‐231‐luc sub‐cellular clones were relatively resistant to HLA‐G CAR‐*γδ*T‐mediated cytotoxic killing compared to their parental cells (Figure 1F). Additionally, the results confirmed that PD‐L1 was upregulated in MDA‐MB‐231 cells in response to the challenge of HLA‐G‐targeted CAR‐*αβ*T and CAR‐NK cells (Figure S1, Supporting Information). On the other hand, PD‐1 expression was upregulated in Nb‐CAR‐expressing *γδ*T as well as *αβ*T and NK cells after coculturing with MDA‐MB‐231 cells (Figure S1B,C, Supporting Information). This finding indicates that the resistant phenotype might not be caused by the loss of the CAR antigen HLA‐G but rather by PD‐L1 upregulation. Therefore, we considered that combining the PD‐L1 targeting strategy with HLA‐G Nb‐CAR might represent an ideal approach to mitigating the possibility of immune escape in tumor cells. To further assess this possibility, the anti‐PD‐L1 antibody atezolizumab was administered into the coculture system, and the results indicated that PD‐L1 blockade enhanced Nb‐CAR‐*γδ*T‐induced cytolysis of all 231‐R sub‐clones (Figure 1F). Furthermore, combination with atezolizumab restored the anti‐tumor activity of Nb‐CAR‐*γδ*T cells to suppress 231‐R3 tumor growth in vivo and thereby extend the survival rate of tumor‐bearing mice (Figure 1G–J). Taken together, we speculate that the expression of PD‐L1 in tumor cells may be a crucial factor for evading the cytotoxicity of Nb‐CAR‐*γδ*T cells.

### 2.2 Expression Patterns of HLA‐G and PD‐L1 in Non‐Small‐Cell Lung Cancer (NSCLC) and Triple Negative Breast Cancer (TNBC) Cells

**Figure 2:**
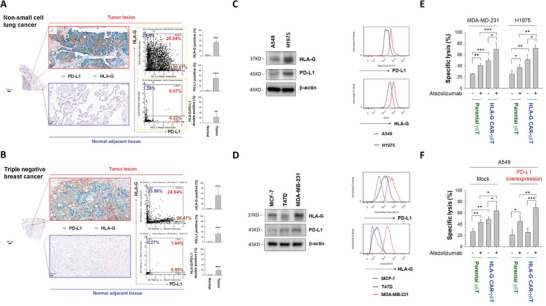
Expression of PD‐L1 in NSCLC and TNBC cells interferes with their sensitivity to HLA‐G targeted CAR‐*γδ*T cells. A,B) HLA‐G and PD‐L1 were frequently expressed in the same lung (*n* = 24) and TNBC (*n* = 30) tumor lesions. HLA‐G and PD‐L1 expressions were determined by 2‐plexed IHC staining using specific antibodies (left). Scale bar = 20 µm of. HLA‐G and PD‐L1 expression levels in tumor lesions and normal adjacent tissues were shown as a tissue cytometry dot‐plot (middle panel) and the quantified data are displayed (right panel). C,D) Expression levels of HLA‐G and PD‐L1 in lung cancer and breast cancer cell lines. C) A549 and H1975; D) MCF‐7, T47D and MDA‐MB‐231 cells were harvested to evaluate the levels of HLA‐G and PD‐L1 by immunoblotting (left panels) and flow cytometry (right panels). E) PD‐L1 blockade re‐enforced cytotoxicity induced by Nb‐CAR‐*γδ*T cells in HLA‐G^high^/PD‐L1^high^ tumor cells. MDA‐MB‐231 and H1975 cells were pretreated with or without 10 µg mL^‐1^ atezolizumab for 15 min and then cocultured with parental or Nb‐CAR‐*γδ*T cells (E:T = 3:1) for 48 h. F) PD‐L1 antagonized HLA‐G targeted CAR‐*γδ*T‐induced cytolysis. Mock or PD‐L1‐overexpressed A549 cells were treated with or without 10 µg mL^−1^ atezolizumab for 15 min and then co‐incubated with parental or Nb‐CAR‐*γδ*T cells (E:T = 3:1) for 48 h. Specific lysis was performed by the LIVE/DEAD cell‐mediated cytotoxicity assay using flow cytometry analysis. These results suggested that elevated PD‐L1 levels on tumor cells may be a risk of immune escape from Nb‐CAR‐*γδ*T therapy in solid tumors. Results are representative of at least three independent experiments. Data represent the mean ± SD, *n* = 3–4, *p < 0.05; **p < 0.01; and ***p < 0.001 based on paired Student's or Student's t‐tests.

Considering that the expression level of PD‐L1 may be a bottleneck for the application of Nb‐CAR‐*γδ*T in the treatment of solid tumors, we analyzed the expression pattern of PD‐L1 and HLA‐G in tumor lesions of NSCLC and TNBC patients. **Figure** **2**A shows that the positive rates of HLA‐G and PD‐L1 expression were approximately 53.5% and 51% in NSCLC tumor samples (*n* = 24), respectively, and 51.9% and 31.9% in TNBC (*n* = 30), respectively (Figure 2B). We also evaluated the expression profile of HLA‐G and PD‐L1 in different NSCLC and TNBC cell lines and found that they were consistent in the different lines and relatively high in NSCLC H1975 and TNBC MDA‐MB‐231 cell lines (Figure 2C,D). We further evaluated the effect of PD‐L1 blockade on Nb‐CAR‐*γδ*T efficacy in the treatment of NSCLC and TNBC cells. In the presence of atezolizumab, the cytotoxicity of Nb‐CAR‐*γδ*T against H1975 and MDA‐MB‐231 cells was significantly enhanced (Figure 2E). Conversely, overexpression of PD‐L1 reduced Nb‐CAR‐*γδ*T‐induced cytolysis, while the addition of atezolizumab restored the Nb‐CAR‐*γδ*T cytotoxicity in PD‐L1 overexpressed A549 cells (Figure 2F). Taken together, we speculate that elevated PD‐L1 levels in solid tumor cells may represent a risk of immune escape for Nb‐CAR‐*γδ*T therapy.

### 2.3 Generation of the BiTE‐Secretable Nb‐CAR Construct for Dual Targeting of HLA‐G and PD‐L1

**Figure 3:**
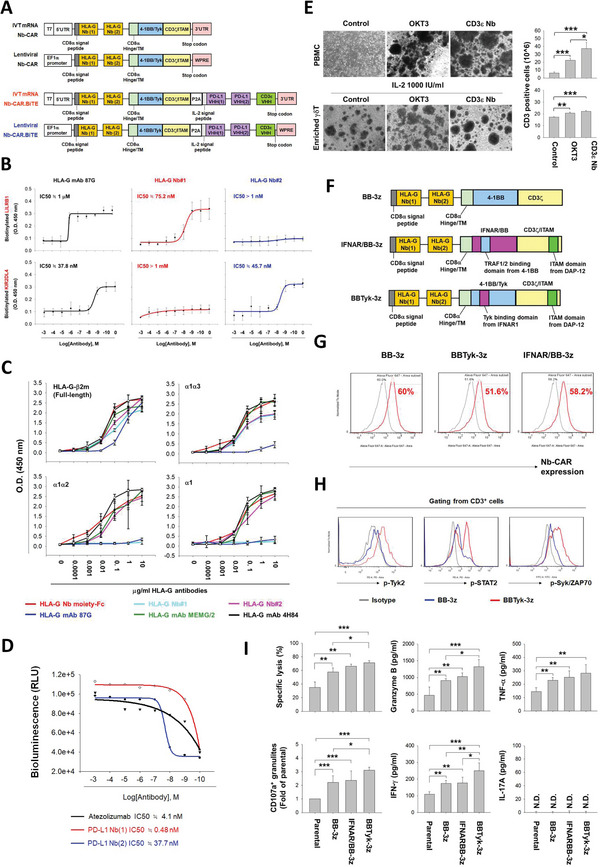
Characterization of the Nb‐CAR.BiTE construct. A) Schematic representation of mRNA and lentiviral HLA‐G Nb‐CAR, with or without combined secretable PD‐L1/CD3*ε*‐targeted Nb‐BiTE constructs. The bi‐epitopic Nb‐CAR comprises two tandem extracellular HLA‐G Nbs fused with a CD8*α* hinge/TM, followed by a modified 4‐1BB cytosolic domain (4‐1BB/Tyk) and a modified CD3*ξ* intracellular domain (CD3*ξ*/ITAM) linked to a self‐cleavage peptide P2A to separate the secretable bivalent Nb‐BiTE, which includes two PD‐L1 Nbs linked to a CD3ɛ Nb. B) The blocking activity of HLA‐G Nbs. The blockade activity of HLA‐G Nb clones #1 and #2 or HLA‐G mAb 87G to LILRB1/HLA‐G (upper panels) and KIR2DL4/HLA‐G (bottom panel) were determined by competitive ELISA. C) HLA‐G Nb moiety capable to bind to all HLA‐G isoforms. The binding activity of HLA‐G Nb clones and it derived bi‐epitopic Nb moiety to recombinant HLA‐G‐*β*2M (corresponding to HLA‐G G1 and G5), HLA‐G *α*1*α*3 domains (G2 and G6), HLA‐G *α*1*α*2 domains (G4) and HLA‐G *α*1 domain (G5) were examined by ELISA‐based binding assay. D) PD‐L1 blockade Nbs. The activity of PD‐L1 Nb clones #1 and #2 was determined by PD‐L1/PD‐1 blockade bioassay. E) Biofunction and affinity of CD3*ε* Nb. PBMCs (1 × 10^6^ cells) were treated with/ without 100 ng CD3*ε* Nb or OKT3. The enriched *γδ*T cells from PBMCs were supplemented with 1000 IL mL^‐1^ IL‐2 and then treated with or without 100 ng CD3*ε* Nb or OKT3. After 6 d, the images were taken (upper panel), Scale bar = 20 µm, and the cell numbers were counted and normalized to the CD3^+^ cell populations (bottom panel). Evaluation of the novel CAR signaling cassette. F) Bi‐epitopic HLA‐G Nb‐CARs were engineered using 4‐1BB‐CD3*ξ* (BB‐3z) or modified signaling cassettes (IFNAR/BB‐3z or BBTyk‐3z). G) BB‐3z‐, IFNAR/BB‐3z‐, or BBTyk‐3z‐based HLA‐G Nb‐CAR lentiviral particles (MOI = 3) were transduced into *γδ*T cells, and after three days, the expression of Nb‐CAR was determined by flow cytometry. H,I) Parental and BB‐3z‐, IFNAR/BB‐3z‐, or BBTyk‐3z‐Nb‐CAR‐*γδ*T cells were cocultured with MDA‐MB‐231 cells at an E:T ratio of 3:1. H) After 2 h, the phosphorylation status of STAT2 and Syk/ZAP70 in the CAR‐*γδ*T cells was determined; and I) after 24 h, the induced cytotoxicity was determined using a LIVE/DEAD cell‐mediated cytotoxicity assay (upper left panel); CD107a expression in CAR‐*γδ*T cells was detected by flow cytometry using specific antibodies (bottom left panel); and their supernatants were harvested to detect the contents of granzyme B, IFN‐*γ*, TNF‐*α*, and IL‐17A by ELISA. These data demonstrated that the elements for engineering the Nb‐CAR.BiTE construct were functional and may be superior than that of the conventional CAR design. Results are representative of at least three independent experiments. Data represent the mean ± SD, n = 3; *p < 0.05; **p < 0.01; and ***p < 0.001 based on paired Student's t‐test.

BiTE targeting PD‐L1 may be a better strategy to avoid safety concerns than CAR‐T targeting PD‐L1.^[^ ^30^, ^31^, ^32^ ^]^ Therefore, we developed a Nb‐CAR.BiTE construct that consists of a bi‐epitopic HLA‐G‐specific Nbs, followed by a CD8*α* hinge, transmembrane domain, modified 4‐1BB intracellular domain (ICD) with an inserted Tyk2‐binding motif excerpted from the interferon‐*α*/*β* receptor 1 (IFNAR1), and CD3*ξ* ICD containing an immunoreceptor tyrosine‐based activation motif (ITAM) extracted from DAP‐12, and it is finally linked with a self‐cleavage peptide P2A with secretable bivalent Nb‐BiTE targeting PD‐L1/CD3 epsilon (CD3*ε*). The extracellular bi‐epitope Nb‐CAR moiety consists of HLA‐G Nb #1 and #2, which are capable of blocking the interaction between HLA‐G and its receptors LILRB1 and KIR2DL4, respectively. The insertion of a Tyk‐binding motif copied from a IFNAR1 fragment into the 4‐1BB ICD is considered to extend the persistence and cytotoxic function of T cells.^[^ ^33^, ^34^ ^]^ The incorporation of an additional ITAM copied from a DAP‐12 fragment into the C‐terminal residues of the CD3*ξ* ICD aims to enhance cytotoxic function with low cytokine production.^[^ ^35^ ^]^ The secretable bivalent PD‐L1 Nb‐BiTE moiety with two different PD‐L1‐targeted Nb clones and a CD3ɛ Nb is designed to maximize the binding affinity to PD‐L1 and engage PD‐L1‐expressing tumor cells with CD3^+^ T cells. The expression of this construct was driven by IVT mRNA or a lentiviral vector strategy (**Figure** **3**A). The biological activity of the major elements comprising the Nb‐CAR.BiTE constructs was investigated. By comparison with conventional antibody fragments, Nb not only offers comparable binding affinity but also flexibility for genetic manipulation due to its smaller size.^[^ ^36^ ^]^ Thus, we selected HLA‐G, PD‐L1, and CD3*ε*‐specific Nb clones by using a camelid naïve Nb phage display library. HLA‐G can be alternatively spliced to four membrane‐bound isoforms (G1‐G4) and three soluble isoforms (G5‐G7), and they all function as immune inhibitory ligands to T, NK, macrophage, and dendritic cells through its receptors, including LILRB1 and KIR2DL4.^[^ ^37^ ^]^ The *α*1 domain (incorporated into all isoforms) is essential for interacting with KIR2DL4 and impairing the functions of T and NK cells.^[^ ^38^, ^39^ ^]^ On the other hand, the *α*3 domain (consisting of G1, G2, G5, and G6) is required for binding to LILRB1 and functions to inhibit T, NK, and B cells, as well as macrophages and dendritic cells.^[^ ^39^, ^40^ ^]^ Therefore, we believed that simultaneous blockade of both the *α*1 and *α*3 domains may be a potent strategy for overcoming the immune‐suppressive function of HLA‐G. As shown in Figure 3B, HLA‐G Nb#1 and #2 could effectively compete for the interaction between HLA‐G and its receptors LILRB1 and KIR2DL4, respectively, which corresponds to the results that HLA‐G Nb#1 could only bind with full‐length and *α*3 domain (LILRB1 binding site)‐containing recombinant HLA‐G isoforms^[^ ^40^ ^]^ while Nb#2 could interact with all HLA‐G isoforms (all isoforms harbor an *α*1 domain containing KIR2DL4 binding motif).^[^ ^38^ ^]^ Accordingly, this bi‐epitope HLA‐G Nb‐CAR is capable of recognizing all HLA‐G isoforms (Figure 3C) and simultaneously blocking the interaction between HLA‐G and its receptors LILRB1 and KIR2DL4. Two PD‐L1 Nbs exhibited PD‐L1/PD‐1 blockade activity (Figure 3D). Additionally, the CD3*ε* Nb promoted CD3^+^ cell proliferation in peripheral blood mononuclear cells (PBMCs) and enriched *γδ*T cells as well (Figure 3E), which were comparable to a commercial antibody OKT3. The bi‐epitopic HLA‐G and PD‐L1 Nb‐BiTE moieties displayed nano‐to‐picomolar binding affinities (Figure S2A–C, Supporting Information). Moreover, the function of the modified ICD (BBTyk‐3z) was characterized by comparison with the conventional unmodified 4‐1BB/CD3*ξ* (BB‐3z) ICD and IFNAR/BB‐3z ICD, which was engineered by the insertion of the TRAF1/2 binding motif copied from a 4‐1BB fragment^[^ ^4^ ^]^ into the IFNAR1 ICD, followed by CD3*ξ*, which was C‐terminally inserted with an additional ITAM copied from a DAP‐12 fragment (IFNAR/BB‐3z) ICD using the same HLA‐G‐targeted Nb‐CAR lentiviral construct that transduces *γδ*T cells (Figure 3F). We found that the BB‐3z‐, BBTyk‐3z‐, and IFNAR/BB‐3z‐based CAR constructs had similar transduction efficiencies in *γδ*T cells (Figure 3G). After coculturing the lentiviral‐transduced *γδ*T cells with MDA‐MB‐231 cells, we found that the BBTyk‐3z‐based Nb‐CAR successfully induced phosphorylation of STAT2 (the downstream mediator of the Tyk‐binding motif from IFNAR1) and Syk/ZAP70 (the downstream of ITAM from DAP‐12 and CD3*ξ*) in *γδ*T cells (Figure 3H). Compared with the BB‐3z‐ and IFNAR/BB‐3z‐based constructs, the BBTyk‐3z‐based CAR construct induced superior cytotoxicity against MDA‐MB‐231 cells and secreted much more IFN‐*γ*, although it did not lead to differences in the expression levels of CD107a^+^ cytolytic granules and secretion of granzyme B and TNF‐*α*. In addition, the extracellular content of IL‐17A, a protumor cytokine produced by *γδ*T cells, was not detectable in these ICD construct‐based CAR‐*γδ*T cocultures (Figure 3I). Furthermore, we also demonstrated that the BBTyk‐3z‐based CAR‐*γδ*T cells also exerted better tumor growth suppression compared with BB‐3z‐based CAR‐*γδ*T cells (Figure S3A–C, Supporting Information). Taken together, we speculated that the utilization of the BBTyk‐3z‐based ICD in the Nb‐CAR.BiTE construct could drive more potent anti‐tumor responses than the conventional BB‐3z‐based ICD.

### 2.4 Characteristics of IVT mRNA‐Driven Nb‐CAR.BiTE‐*γδ*T Cells

**Figure 4:**
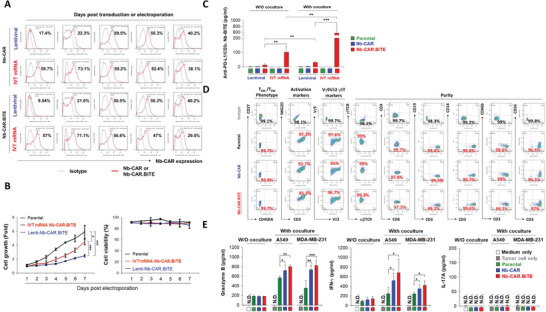
Characterization of mRNA‐driven Nb‐CAR.BiTE‐*γδ*T cells. A) Nb‐CAR expression was assessed after lentiviral transduction or mRNA electroporation. *γδ*T cells (1 × 10^6^) were electroporated with 2 µg Nb‐CAR/CAR.BiTE IVT mRNA or transduced with lentiviral Nb‐CAR/CAR.BiTE particles (MOI = 3). The Nb‐CAR expression on the indicated days was determined through flow cytometry using a specific antibody against VHH. B,C) Superior cell growth and Nb‐BiTE secretion using the mRNA delivery strategy. B) Cell number and viability were recorded during 7 d after transfection of the mRNA or lentiviral Nb‐CAR.BiTE transgene. C) Contents of PD‐L1‐targeted Nb‐BiTE in the supernatants from mRNA‐ or lentiviral‐driven Nb‐CAR.BiTE‐*γδ*T cell cultures were detected by an ELISA‐based coating with recombinant full‐length PD‐L1. D) Phenotype and purity of mRNA‐driven Nb‐CAR.BiTE‐*γδ*T cells. T effector and central memory phenotype, activating marker, and purity of Nb‐CAR.BiTE mRNA‐electroporated *γδ*T cells were analyzed through flow cytometry using specific antibodies against CD27, CD45RA, CD3, NKG2D, V*δ*2, V*γ*9, *αβ*TCR, *γδ*TCR, CD4, CD8, CD19, CD14, CD66b, or CD56. Isotype staining was used as a gating control for positive staining. E) Increased secretion of granzyme B and IFN‐*γ*, but not IL‐17A, from Nb‐CAR.BiTE‐*γδ*T cells after coculture with A549 or MDA‐MB‐231 cells. The contents of granzyme B (upper panel), IFN‐*γ* (middle panel), and IL‐17A (bottom panel) in culture supernatants were measured using ELISA. These results showed that mRNA‐driven Nb‐CAR.BiTE‐*γδ*T cells would be an effective strategy for the manufacture of Nb‐CAR.BiTE‐*γδ*T cells. Results are representative of at least three independent experiments. Data represent the mean ± SD, *n* = 3–4, *p < 0.05; **p < 0.01; and ***p < 0.001 based on paired Student's t‐tests.

We next evaluated the expression and characteristics of IVT mRNA‐driven Nb‐CAR in *γδ*T cells. Compared to lentiviral particle transduction, IVT mRNA delivered by electroporation rapidly drove Nb‐CAR expression with higher expansion activity and Nb‐BiTE secretion (**Figure** **4**A–C). The phenotypic characterization of the mRNA‐electroporated Nb‐CAR.BiTE‐*γδ*T cells showed that they exhibited 99% effector memory T‐cells (CD27^neg^/CD45RA^neg^) and more than 95% NKG2D expression. In addition, the population of V*γ*9V*δ*2 *γδ*T cells and *γδ*TCR^+^/*αβ*TCR^−^ cell were approximately 95% and more than 99%, respectively (Figure 4D). Furthermore, after challenge with A549 and MDA‐MB‐231 cells, mRNA‐driven Nb‐CAR.BiTE‐*γδ*T cells had higher secretion of granzyme B and IFN‐*γ* than the parental and mRNA‐driven Nb‐CAR‐*γδ*T groups, while the secretion of IL‐17A was undetectable (Figure 4E). Moreover, we observed that both mRNA‐ and lentiviral‐engineered Nb‐CAR.BiTE‐*γδ*T cells exerted similar anti‐tumor activity in vivo (Figure S4, Supporting Information). Accordingly, IVT mRNA delivered by electroporation would be an effective strategy for the manufacture of Nb‐CAR.BiTE‐*γδ*T cells.

### 2.5 PD‐L1‐Targeting Nb‐BiTE Secreted from mRNA‐Engineered Nb‐CAR.BiTE‐*γδ*T Triggers Bystander Effector Cells against Tumor Cells

**Figure 5:**
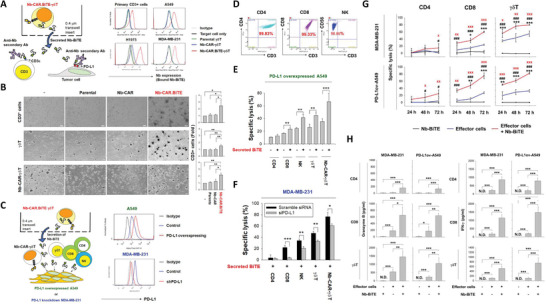
Evaluation of Nb‐BiTE secreted from Nb‐CAR‐*γδ*T cells. A–D) Functional binding of Nb‐BiTE released from Nb‐CAR‐*γδ*T cells. A) Diagram shows the cell non‐contact coculture system for Nb‐CAR.BiTE‐*γδ*T cells and target cells (left panel). Secreted Nb‐BiTE from Nb‐CAR‐*γδ*T cells was detectable on target cells. We seeded 1 × 10^5^ isolated primary CD3‐positive cells from PBMCs, A549, H1975, or MDA‐MB‐231 cells on the bottom, with or without exposure to 5 × 10^5^ Nb‐CAR.BiTE‐*γδ*T cells on the top, in impenetrable wells for 24 h. Subsequently, the bottom cells were harvested and Nb‐BiTE signals were detected by flow cytometry using specific antibody against VHH (right panel). B) Secreted Nb‐BiTE from Nb‐CAR‐*γδ*T cells promoted CD3‐positive cell proliferation. We cocultured 1 × 10^6^ isolated primary CD3^+^ cells, parental, or Nb‐CAR‐*γδ*T cells with or without 1 × 10^6^ parental, Nb‐CAR, or Nb‐CAR.BiTE‐*γδ*T cells on the top well for 6 days. Subsequently, the CD3^+^ cell numbers were normalized to the non‐coculture group. C) Nb‐BiTE released from Nb‐CAR‐*γδ*T cells strengthened effector cells against PD‐L1‐expressing tumor cells. Schematic illustration of the non‐contact coculture system for Nb‐CAR.BiTE‐*γδ*T cells, which release Nb‐BiTE to engage CD3^+^ effectors and PD‐L1‐overexpressing tumor cells (left panel). The PD‐L1 level on PD‐L1‐overexpressing A549 and PD‐L1 knockdown MDA‐MB‐231 stable clones were examined by flow cytometry analysis (right panel). D) Purity of the isolated CD4‐ and CD8‐positive and CD3‐/CD56^+^ cells from PBMCs were checked by flow cytometry. E) We cocultured 1 × 10^5^ PD‐L1‐overexpressing A549 cells with an individual healthy donor‐derived CD4^+^, CD8^+^, NK, parental *γδ*T, or Nb‐CAR‐*γδ*T cells on the bottom well and with or without 5 × 10^5^ Nb‐CAR.BiTE‐*γδ*T cells on the top well for 72 h. F) PD‐L1 determined the cell‐killing ability of effector cells when exposed to released Nb‐BiTE. PD‐L1‐stable knockdown or scramble‐control MDA‐MB‐231 cells (1 × 10^5^) were cocultured with an individual healthy donor‐derived CD4^+^, CD8^+^, NK, parental *γδ*T, or Nb‐CAR‐*γδ*T cells on the bottom well and with 5 × 10^5^ Nb‐CAR.BiTE‐*γδ*T cells on the top well for 72 h. G,H) PD‐L1‐targeted Nb‐BiTE enhanced anti‐tumor responses of T cells. MDA‐MB‐231 or PD‐L1‐overexpressing A549 cells were cocultured with or without the isolated CD4^+^ or CD8^+^ cells at an E:T ratio of 5:1; or *γδ*T cells at an E:T ratio of 3:1 in the presence/absence of recombinant Nb‐BiTE (1 ng mL^‐1^). G) After 24, 48, or 72 h of coculture, we determined the cytotoxicity induced by these effector cells. H) After 48 h of coculture, the supernatants were harvested for detecting the contents of granzyme B and IFN*γ* by ELISA. Specific lysis was determined using LIVE/DEAD cell‐mediated cytotoxicity assay. These results suggested that the secreted PD‐L1 targeting Nb‐BiTE from Nb‐CAR.BiTE‐*γδ*T could trigger bystander effector cells active against PD‐L1‐expressing tumor cells. Results are representative of at least three independent experiments. Data represent the mean ± SD, *n* = 4, *,^#^,^X^ *p* < 0.05; **,^##^,^XX^ *p* < 0.01; and ***,^###^,^XX X^ *p* < 0.001 based on paired Student's t‐test. For sub‐Figure G, * represents significant differences between Nb‐BiTE and effector cells (Nb‐BiTE vs effector cells); # represents significant differences between Nb‐BiTE and effector cells+Nb‐BiTE; ^X^ represents significant differences between effector cells and effector cells+ Nb‐BiTE.

To assess the function of the secreted Nb‐BiTE, a transwell assay was used as a cell non‐contact coculture system (**Figure** **5**A, left panel) as previously described.^[^ ^30^ ^]^ Flow cytometry analysis showed that Nb fragments were detected on isolated primary CD3‐positive cells as well as on A549, H1975, and MDA‐MB‐231 cells (Figure 5A, right panel). After coculture with mRNA‐driven Nb‐CAR.BiTE‐*γδ*T cells, the proliferation of CD3^+^ cells was significantly increased in PBMCs, non‐transfected *γδ*T, and Nb‐CAR‐*γδ*T cells (Figure 5B). Moreover, the cytolysis proportion of PD‐L1‐overexpressed A549 cells induced by CD8^+^ T, NK, non‐transfected *γδ*T, and Nb‐CAR‐*γδ*T cells was significantly enhanced after exposure to mRNA‐driven Nb‐CAR.BiTE‐*γδ*T in the non‐contact coculture system (Figure 5C–E). In contrast, the enhancement caused by secreted Nb‐BiTE was disrupted by PD‐L1 silencing in MDA‐MB‐231 cells (Figure 5F). Furthermore, we added the purified recombinant Nb‐BiTE into the CD4 (or CD8, or *γδ*T)/MDA‐MB‐231(or PD‐L1‐overexpressing A549) cocultures and analyzed the induced cytotoxicity and cytokine responses to demonstrate that PD‐L1 Nb‐BiTE is an authentic T cell engager. We found that the addition of recombinant Nb‐BiTE not only enhanced the cytotoxic killing ability of the isolated CD4^+^ and CD8^+^ cells as well as *γδ*T cells (Figure 5G) but also increased the secretion of granzyme B and IFN‐*γ* after coculturing with MDA‐MB‐231 or PD‐L1‐overexpressing A549 cells (Figure 5H). Taken together, these findings indicate that Nb‐BiTE is secreted by mRNA‐engineered Nb‐CAR.BiTE‐*γδ*T cells could bind to its targets and thus promote engagement between effector and target cells, which subsequently activated the cytotoxic killing responses.

### 2.6 mRNA‐Driven Nb‐CAR.BiTE‐*γδ*T Is Effective against HLA‐G and/or PD‐L1 Positive Tumor Cells

**Figure 6:**
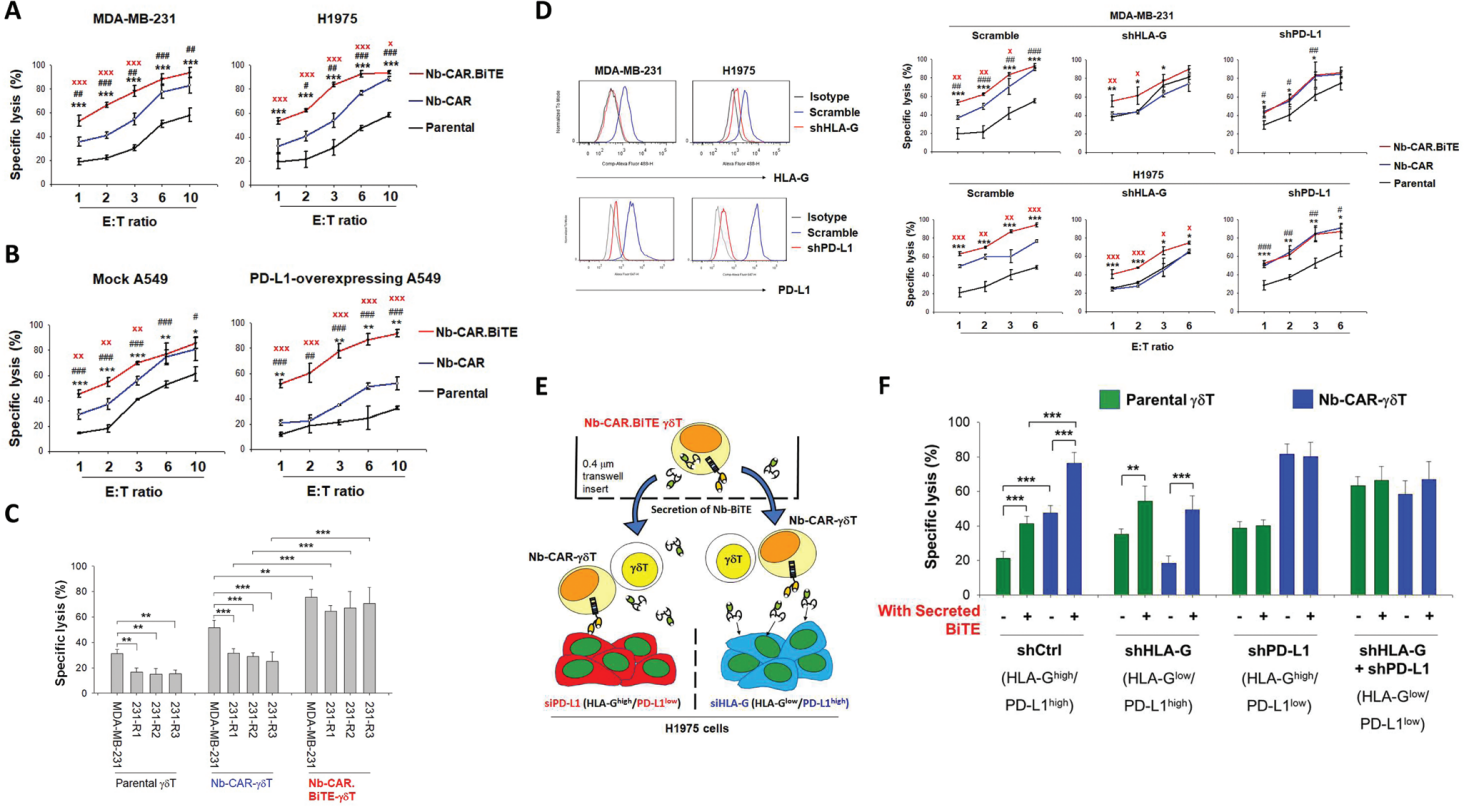
Nb‐CAR.BiTE‐*γδ*T cells are effective to treat tumor cells with HLA‐G and/or PD‐L1 expression in vitro. A,B) Nb‐CAR.BiTE‐*γδ*T cells effectively ablated HLA‐G^+^ and/or PD‐L1‐overexpressed tumor cells. Parental, Nb‐CAR, or Nb‐CAR.BiTE‐*γδ*T cells were cocultured with 1 × 10^5^ MDA‐MB‐231 or H1975 cells A) mock or PD‐L1‐stably overexpressing A549 cells B) at an E:T ratio of 1:1–10:1 for 72 h. Induced cytotoxicity was measured by LIVE/DEAD cell‐mediated cytotoxicity assay C) Nb‐CAR.BiTE‐*γδ*T cells have potent cytotoxicity against the PD‐L1‐overexpressing immune escape variants. We cocultured 1 × 10^5^ parental MDA‐MB‐231, 231‐R1, ‐R2 or ‐R3 cells with Parental, Nb‐CAR, or Nb‐CAR.BiTE‐*γδ*T cells at an E:T ratio of 3:1 for 72 h. Specific lysis was determined using LIVE/DEAD cell‐mediated cytotoxicity assay. D) *γδ*T effector cells retain their natural anti‐tumor effect on tumor cells with low levels of CAR or BiTE antigen expression. HLA‐G and PD‐L1 levels in H1975 and MDA‐MB‐231 cells were detected through flow cytometry following HLA‐G or PD‐L1 stable knockdown (left panel). HLA‐G‐ or PD‐L1‐knockdown H1975 and MDA‐MB‐231 cells (1 × 10^5^) were co‐incubated with parental, Nb‐CAR‐expressing, or Nb‐CAR.BiTE‐expressing *γδ*T cells at an E:T ratio of 1:1, 2:1, 3:1, and 6:1 for 72 h. Subsequently, specific lysis of target cells was determined by a LIVE/DEAD cell‐mediated cytotoxicity assay. E,F) PD‐L1 on tumor cells determines the sensitivity of secreted Nb‐BiTE, and both HLA‐G and PD‐L1 expression affect the cytolysis induced by *γδ*T cells. E) Schematic illustration of non‐contact coculture system for Nb‐CAR.BiTE‐*γδ*T cells, which release Nb‐BiTE to engage *γδ*T cells against H1975 cells. F) H1975 cells were transfected with or without siHLA‐G and/or siPD‐L1 for 48 h, then seeded in the bottom wells (1 × 10^5^ cells per well), and then cocultured with parental or Nb‐CAR‐*γδ*T cells (E:T = 1:1) with or without Nb‐CAR.BiTE‐*γδ*T cells in the top transwell inserts (5 × 10^5^ cells per insert) for 72 h. Subsequently, the specific lysis of target cells was examined using a LIVE/DEAD cell‐mediated cytotoxicity assay. These results suggested that Nb‐CAR.BiTE‐*γδ*T cells are effective to against HLA‐G and/or PD‐L1 positive tumor cells even antigens are low expressed. Results are representative of at least three independent experiments. Data represent the mean ± SD, *n* = 4, *,^#^,^X^ *p* < 0.05; **,^##^,^XX^ *p* < 0.01; ***,^###^,^XX X^ *p* < 0.001; stars represent significant differences between parental and Nb‐CAR.BiTE‐*γδ*T; pound signs represent significant differences between parental and Nb‐CAR‐*γδ*T; and double daggers represent significant differences between Nb‐CAR‐*γδ*T and Nb‐CAR.BiTE‐*γδ*T based on paired Student's t‐tests.

Subsequently, we evaluated the cytotoxic effect of mRNA‐driven Nb‐CAR.BiTE‐*γδ*T cells on solid tumor cells with various PD‐L1 and HLA‐G levels. Compared with parental *γδ*T and Nb‐CAR‐*γδ*T cells, Nb‐CAR.BiTE‐*γδ*T cells more efficiently induced cytolysis of MDA‐MB‐231 and H1975 cells with intrinsically high levels of HLA‐G and PD‐L1 (HLA‐G^high^/PD‐L1^high^) (**Figure** **6**A). Additionally, Nb‐CAR.BiTE‐*γδ*T cells exhibited higher cytolytic efficiency than Nb‐CAR *γδ*T and parental *γδ*T cells when challenged against PD‐L1‐overexpressed A549 cells (Figure 6B). Furthermore, Nb‐CAR.BiTE‐*γδ*T could effectively eliminate the viability of 231‐R1, R2, and R3 cells (Figure 6C). Moreover, the cytotoxic capacity of Nb‐CAR‐*γδ*T cells was attenuated against HLA‐G knockdown MDA‐MB‐231 and H1975 cells but elevated against PD‐L1 knockdown cells. In contrast, the cytotoxicity of Nb‐CAR.BiTE‐*γδ*T against PD‐L1‐silencing cells was reduced to a similar level to that of Nb‐CAR‐*γδ*T cells. Moreover, although HLA‐G or PD‐L1 silencing increased the susceptibility of cancer cells to cytolysis by parental *γδ*T cells, both Nb‐CAR‐*γδ*T and Nb‐CAR.BiTE‐*γδ*T cells still had superior cell‐killing activity compared to the parental *γδ*T cells (Figure 6D). We further evaluated the effect of secreted Nb‐BiTE on antigen‐low target cells by using the non‐contact coculture system (Figure 6E). Under exposure to mRNA‐driven Nb‐CAR.BiTE‐*γδ*T cells, there was no additional increase in cytolysis induced by parental *γδ*T and Nb‐CAR‐*γδ*T cells in the PD‐L1‐knockdown H1975 cells. Moreover, parental and Nb‐CAR‐*γδ*T cells exerted similar cytotoxicity against HLA‐G/PD‐L1 double‐knockdown H1975 cells, even under the exposure to Nb‐CAR.BiTE‐*γδ*T cells (Figure 6F). These findings not only showed that cancer cells can avoid the natural antitumor activity of *γδ*T cells by regulating HLA‐G and PD‐L1 but also suggest that the secreted Nb‐BiTE could serve as a component to enhance the antitumor activity of *γδ*T cells against PD‐L1^+^ cancer cells.

### 2.7 mRNA‐Driven Nb‐CAR.BiTE‐*γδ*T Is Effective against PD‐L1‐Overexpressing Solid Tumors In Vivo

**Figure 7:**
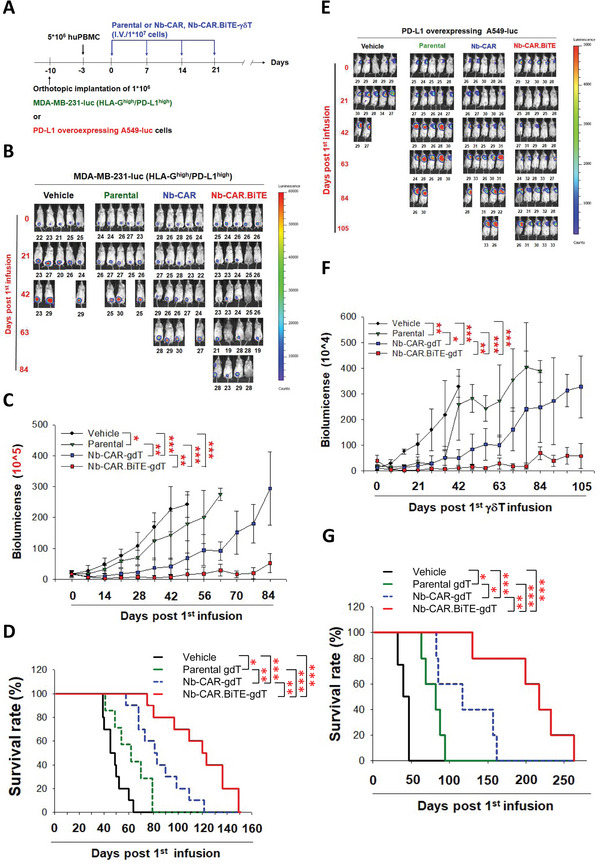
Anti‐tumor activity of Nb‐CAR.BiTE‐*γδ*T cells in the orthotopic PBMC‐CDX‐NSG mouse models. A) Protocol for evaluating the anti‐tumor activity of mRNA‐driven Nb‐CAR.BiTE‐*γδ*T cells in the PBMC‐CDX‐NSG mice bearing orthotopic HLA‐G^high^/PD‐L1^high^ TNBC or PD‐L1 overexpressing lung tumors. Seven days after inoculation with 1 × 10^6^ tumor cells, the mice received tail vein injection with PBMCs (5 × 10^6^ cells per mouse). Three days later, the parental, Nb‐CAR or Nb‐CAR.BiTE‐*γδ*T cells (1 × 10^7^ cells/mouse) were injected through tail vein once a week for 4 weeks. B‐G) Superior anti‐tumor activity of Nb‐CAR.BiTE‐*γδ*T cells in the PBMC‐CDX‐NSG mouse model. The tumor growth of the orthotopically inoculated B,C) MDA‐MB‐231 (*n* = 10) and E,F) PD‐L1‐overexpressing A549 cells (*n* = 5) were monitored weekly through IVIS. D,G) Survival rates were recorded. These results suggested that Nb‐CAR.BiTE‐*γδ*T is effective against HLA‐G/PD‐L1‐double positive solid tumors as well as PD‐L1‐overexpressing solid tumors in vivo. Results are representative of three independent experiments. Data represent the mean ± SD; *p < 0.05; **p < 0.01; and ***p < 0.001 based on the Kaplan–Meier method and log‐rank test.

PBMC‐humanized NSG mice bearing cell line‐derived xenografted (CDX) tumors (PBMC‐CDX‐NSG) have been proposed for evaluating the in vivo efficiency of BiTEs, especially those secreted from CAR‐T cells.^[^ ^19^, ^41^ ^]^ **Figure** **7**A shows the experimental protocol for the efficiency of mRNA‐driven Nb‐CAR.BiTE‐*γδ*T cells in the PBMC‐CDX‐NSG murine model bearing MDA‐MB‐231 (intrinsic HLA‐G^high^/PD‐L1^high^) or PD‐L1‐overexpressing A549 tumors. The infusion of mRNA‐engineered Nb‐CAR.BiTE‐*γδ*T cells effectively suppressed the growth of MDA‐MB‐231 inoculated tumors and prolonged the survival rate of mouse compared with parental *γδ*T and Nb‐CAR‐*γδ*T infusion groups (*n* = 10) (Figure 7B–D). Identically, mRNA‐driven Nb‐CAR.BiTE‐*γδ*T also exerted superior anti‐tumor efficiency compared with parental *γδ*T and Nb‐CAR‐*γδ*T infusion groups in the PD‐L1‐overexpressing A549 tumor‐bearing mouse model (*n* = 5) (Figure 7E–G). In addition, we also demonstrated that the infusion of Nb‐CAR.BiTE‐*γδ*T cells more efficiently suppressed xenografted MDA‐MB‐231 tumor growth compared with Nb‐CAR‐*γδ*T cells combined with the systemic administration of atezolizumab in vivo (Figure S4, Supporting Information). Moreover, we observed that both mRNA‐ and lentiviral‐engineered Nb‐CAR.BiTE‐*γδ*T cells exerted similar anti‐tumor activity in vivo (Figure S5, Supporting Information). These results suggested that combining Nb‐CAR and Nb‐BiTE into single platform driven by a bicistronic mRNA construct would be more effective against solid tumors in vivo.

### 2.8 Expression of mRNA‐Driven Nb‐CAR Promotes Infiltration of *γδ*T Cells into Tumor Sites

**Figure 8:**
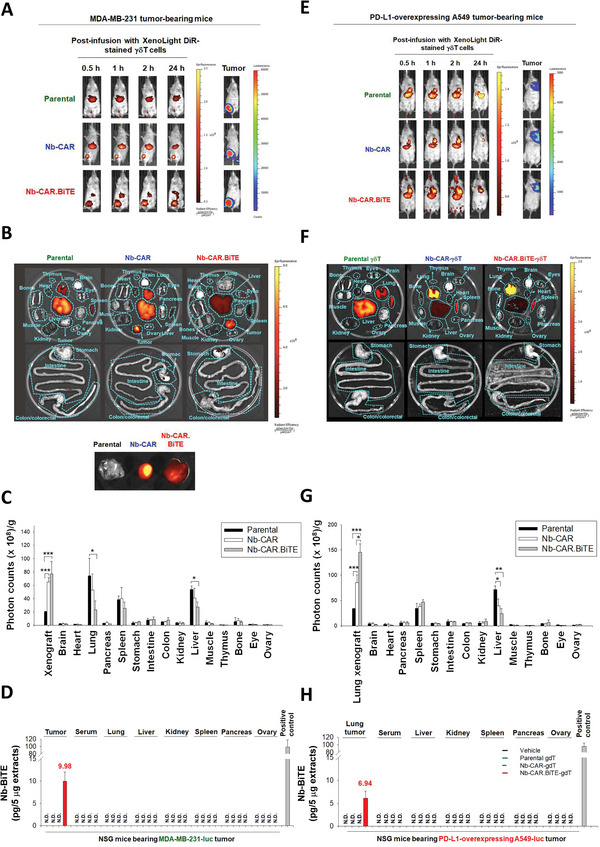
Biodistribution of Nb‐CAR‐expressing *γδ*T cells in vivo. A‐C) Nb‐CAR expression enhanced *γδ*T cells accumulated in the local breast tumor site. XenoLight DiR dye‐stained parental, Nb‐CAR or Nb‐CAR.BiTE‐*γδ*T cells (1 × 10^7^ cells per mouse) were infused into MDA‐MB‐231 tumor‐bearing NSG mice (*n* = 5) via tail vein injection. A) Fluorescent signals of each mouse were monitored by IVIS at the indicated time points. B,C) Mice were sacrificed after 24 h, B) organs and tumor tissues were harvested for measuring fluorescent signals by IVIS, and C) the quantitative results were identified and normalized per gram of tissue samples. D) Nb‐BiTE was only detectable in inoculated tumors of Nb‐CAR.BiTE‐*γδ*T‐treated mice bearing MDA‐MB‐231 tumors, but not in other organs. The contents of PD‐L1/CD3*ε* Nb‐BiTE in the tissue extracts (5 µg) were determined by ELISA‐based method coating with PD‐L1 recombinant protein. The supernatants of Nb‐CAR.BiTE‐*γδ*T cell cultures were used as a positive control. E–G) Nb‐BiTE‐secreting Nb‐CAR‐*γδ*T cells increased the accumulation of infused *γδ*T cells at the PD‐L1‐overexpressing tumor inoculation site. PD‐L1‐overexpressing A549 tumor‐bearing mice were injected with XenoLight DiR dye‐stained parental, Nb‐CAR, or Nb‐CAR.BiTE‐*γδ*T cells (1 × 10^7^ cells per mouse). E) The fluorescent signals were monitored by IVIS at the indicated time points. F,G) 24 h after *γδ*T cell infusion, the mice were sacrificed and fluorescent signals of the organs and tumor tissues were determined by IVIS (F) and quantified (G). H) Presence of Nb‐BiTE was restricted in PD‐L1‐overexpressing tumor inoculation lung tissues but not in other organs in vivo. The tissue extracts were harvested and 5 µg of each sample were analyzed the expression of PD‐L1/CD3*ε* Nb‐BiTE by ELISA‐based method with the coating of PD‐L1 recombinant protein. The supernatants of Nb‐CAR.BiTE‐*γδ*T cell cultures were used as a positive control. Results are representative of at least three independent experiments. Data represent the mean ± SD, *n* = 3, *p < 0.05; **p < 0.01; and ***p < 0.001; paired Student's t‐test.

We next investigated the biodistribution and the tumor trafficking ability of *γδ*T cells in PBMC‐CDX‐NSG mice following the infusion of mRNA‐engineered Nb‐CAR‐*γδ*T or Nb‐CAR.BiTE‐*γδ*T cells. The increased localization of Nb‐CAR‐*γδ*T and Nb‐CAR.BiTE‐*γδ*T cells were observed at the local tumor sites in the MDA‐MB‐231 tumor‐bearing NSG mouse model (*n* = 5) (**Figure** **8**A–C). In addition, Nb‐BiTE was only detected in MDA‐MB‐231 inoculated tumor extracts and not in the serum and vital organs (Figure 8D). Consistently, the accumulation of mRNA‐driven Nb‐CAR‐*γδ*T cells was observed in the lungs of mice transplanted with PD‐L1‐overexpressing A549 cells (*n* = 5), while the infused mRNA‐driven Nb‐CAR.BiTE‐*γδ*T cells were more concentrated in the lung tissues (Figure 8E–G). In parallel, PD‐L1‐targeted Nb‐BiTE was only detected in lung tissue extracts in mice transplanted with PD‐L1‐overexpressing A549 cells (Figure 8H). The persistence of the infused mRNA‐engineered Nb‐CAR.BiTE‐*γδ*T cells were also evaluated, and the results showed that the amount of *γδ*T cells concentrated in MDA‐MB‐231 tumors began to decrease 3 d after infusion (Figure S6A–D, Supporting Information) while the amount concentrated in the liver and spleen began to increase within 2 and 3 d after infusion, respectively, thus indicating that the liver and spleen represent the homing organs of unmodified *γδ*T cells in mice.^[^ ^42^ ^]^ The *γδ*T cells accumulated in tumor foci were detectable at up to 240 hours post‐infusion, although the signal declined substantially at that time point. Moreover, the secreted Nb‐BiTE was only detectable in tumor extracts within 3 d after infusion of mRNA‐driven Nb‐CAR.BiTE‐*γδ*T cells (Figure S6E, supporting Information). Furthermore, more TCR*δ*‐positive cells were observed in MDA‐MB‐231 tumor lesions of the mRNA‐engineered Nb‐CAR and Nb‐CAR.BiTE‐*γδ*T‐infused mice than in the control and parental *γδ*T‐treated groups. The infusion of Nb‐CAR.BiTE‐*γδ*T cells promoted the infiltration of CD4^+^ and CD8^+^ T cells into tumors (Figure S7, Supporting Information). These results indicate that HLA‐G‐targeted Nb‐CAR potentiates *γδ*T cells to infiltrate into tumors and secreted Nb‐BiTE redirects *γδ*T as well as bystander immune cells into tumor lesions.

### 2.9 mRNA‐Driven Nb‐CAR.BiTE‐*γδ*T Has No Obvious Toxicity In Vitro and In Vivo

To elucidate the toxicity risk of HLA‐G‐targeted therapy in clinical applications, we evaluated the safety of mRNA‐engineered Nb‐CAR.BiTE‐*γδ*T cells in vitro and in vivo. As shown in Figure S8A (Supporting Information), Nb‐CAR.BiTE‐*γδ*T cells did not induce significant cytotoxicity against various types of human primary tissue cultures (2 donors of each tissue type) including lung epitheliums, hepatocytes and pancreatic islets. In contrast, Nb‐BiTE neither increased the cytotoxicity of *γδ*T cells against PD‐L1‐expressing primary normal cells nor modulated the extracellular content of granzyme B and IFN‐*γ* (Figure S8B–D, Supporting Information). Because the functional HLA‐G homolog Qa2 in mouse has contradictory effects with human HLA‐G and exhibits insufficient amino acid identity,^[^ ^43^, ^44^ ^]^ our HLA‐G Nbs may not recognize Qa2 to reflect the toxicity of Nb‐CAR.BiTE‐*γδ*T in murine model. Thus, we used HLA‐G transgenic mice (CBA/Ca‐Tg(H2‐K‐HLA‐G,B2M)1Alm/CmwJ) that exhibit tissue‐specific HLA‐G expression patterns consistent with humans^[^ ^39^, ^45^ ^]^ to assess the toxicity of mRNA‐driven Nb‐CAR.BiTE‐*γδ*T (male and female, *n* = 5 of each gender). The results showed that repeated infusions of Nb‐CAR.BiTE‐*γδ*T did not affect body weight, food intake, activity, presence of diarrhea, and mouse grimace scale (MGS) scores in both male and female mice (Figure S9A,B, Supporting Information). More importantly, TUNEL‐positive apoptotic cells were not significantly increased, even in HLA‐G‐detectable and Nb‐CAR.BiTE‐*γδ*T cells in accumulating organs (Figures S9C–E and S10A–C, Supporting Information). Taken together, the use of mRNA‐driven Nb‐CAR.BiTE‐*γδ*T cell therapy for solid tumors should be effective and presents minimal safety concerns.

## 3 Discussion

The development of CAR‐T and BiTE therapies has led to a new era in cancer care. However, breakthroughs remain to be made in the application of these therapies for the treatment of solid tumors. In the present study, we aimed to address the immune escape caused by PD‐L1 to reinforce the anti‐tumor activity of HLA‐G‐targeted CAR‐T therapy. We developed a single mRNA construct containing an Nb‐based CAR followed by a secretable Nb‐BiTE for dual‐targeting ICP molecules HLA‐G and PD‐L1. V*δ*2 *γδ*T cells represented effector cells and were engineered via electroporation with this Nb‐CAR.BiTE mRNA for the treatment of solid tumors. The results suggest that this strategy could effectively eliminate solid tumors expressing various levels of HLA‐G and/or PD‐L1, especially those with double positive or even overexpression of HLA‐G and PD‐L1. The secreted Nb‐BiTE could promote the activation of Nb‐CAR‐*γδ*T cells, un‐transduced‐*γδ*T cells, and other bystander effector cells against PD‐L1‐expressing tumor cells. The in vivo experiments provided further evidence that engineering with HLA‐G‐targeted Nb‐CAR redirected *γδ*T cells to tumor lesions and the secreted PD‐L1 Nb‐BiTE recruited T cells to infiltrate and accumulate in the local tumor foci. In addition, Nb‐CAR.BiTE‐*γδ*T cells did not cause observable toxicity in either primary human tissue cultures or HLA‐G transgenic mice. Collectively, the significant findings in the present study indicate that mRNA‐driven Nb‐CAR.BiTE‐*γδ*T therapy is a potent strategy that not only specifically target solid tumors with antigen but also recruits bystander effector cells via Nb‐BiTE to attack ICP‐expressing and even antigen‐inadequate cancer cells.

PD‐L1 upregulation in cancer cells has broad effects and represents the mechanism underlying resistance to CAR‐based therapy.^[^ ^46^ ^]^ On the other hand, PD‐1 upregulation in CAR‐T cells results in T cell exhaustion and loss of function.^[^ ^12^ ^]^ Therefore, blockade of PD‐L1/PD‐1 axis has been suggested to improve the potency of CAR‐T therapy.^[^ ^10^, ^25^ ^]^ In addition to anti‐PD‐L1 or anti‐PD‐1 neutralizing antibodies, PD‐1‐knockout (KO) CAR‐T strategies have begun to be developed.^[^ ^16^, ^47^, ^48^ ^]^ In this study, we pioneered the introduction of PD‐L1‐targeting Nb‐BiTE into an HLA‐G Nb‐CAR construct to alleviate immune escape caused by PD‐L1/PD‐1 upregulation during CAR‐T therapy. We believe that this strategy will represent an improvement over previous approaches based on several factors. First, antigen‐targeted CAR‐T infiltrating tumor lesions to locally secrete PD‐L1‐targeting Nb‐BiTE would be more effective with fewer safety concerns than systemic administration of PD‐L1 blockade antibodies.^[^ ^31^ ^]^ Second, the secreted BiTE could recruit bystander effectors and the CD3*ε*‐targeted Nb moiety of BiTE could further activate these effector cells to synergize with Nb‐CAR‐*γδ*T cells against PD‐L1‐expressing tumor cells. Moreover, our data also indicated that the secreted Nb‐BiTE also could disrupt PD‐L1‐mediated inhibition to reinforce NK‐induced cytolysis to tumor cells as well.^[^ ^49^ ^]^ Accordingly, embedding a secretable PD‐L1‐targeting Nb‐BiTE into the Nb‐CAR construct should overcome PD‐L1‐mediated resistance to CAR‐T therapy and potentiate the efficacy of HLA‐G‐targeted Nb‐CAR to combat solid tumors. Additionally, we considered that the elevated B7‐H3 might also play a role in the development of resistance to HLA‐G Nb‐CAR‐*γδ*T cells and promotion of immune escape from PD‐L1 Nb‐BiTE and could induce natural anti‐tumor effects in *γδ*T cells since the tumors were not eradicated by infusion with mRNA‐driven Nb‐CAR.BiTE‐*γδ*T cells in vivo. The involvement of B7‐H3 in our setting should be investigated in future studies.

Because our Nb‐BiTE contains only an anti‐CD3*ε* Nb for stimulating T cells, we could not rule out the possibility that exhaustion would occur in vivo due to the lack of cross‐linking within anti‐CD28. A recent study has indicated that treatment‐free intervals can ameliorate T‐cell exhaustion induced by continuous BiTE treatment.^[^ ^50^ ^]^ We found that the secreted Nb‐BiTE was only detectable in tumor extracts within 3 d after infusion of mRNA‐driven Nb‐CAR.BiTE‐*γδ*T cells (Figure S6E, Supporting Information), implying that Nb‐BiTE stimulation might occur over a 2–3 d interval according to our animal experiment procedures. The results suggest that the transiently secreted Nb‐BiTE from mRNA‐engineered *γδ*T cells might not cause irreversible exhaustion phenotype to impair anti‐tumor activity of *γδ*T cells in vivo. Therefore, we also considered that the length of treatment‐free intervals of our mRNA‐driven Nb‐CAR.BiTE‐*γδ*T cells should be determined prior to its use in clinical studies.

V*δ*2 *γδ*T cells present allogeneic potential,^[^ ^29^ ^]^ and tumor‐infiltrating V*δ*2 *γδ*T cells are associated with favorable outcomes in multiple cancer types.^[^ ^51^ ^]^ The particular antigen‐presenting capacity of V*δ*2 *γδ*T may provide therapeutic benefits through stimulating adaptive immunity.^[^ ^25^, ^26^ ^]^ On the other hand, the intrinsic anticancer activity of V*δ*2 *γδ*T is independent of CAR and MHC,^[^ ^27^ ^]^ which may complement our CAR.BiTE system against tumor cells with insufficient antigens. Consistent with this, we confirmed that un‐engineered V*δ*2 *γδ*T cells are still potent cytotoxic to HLA‐G and/or PD‐L1‐silenced tumor cells (Figure 6F). Moreover, the Nb‐CAR.BiTE‐engineered *γδ*T cells did not secrete IL‐17A, even after engaging with tumor cells (Figure 3F), suggesting that their potential protumor effect could be ruled out.^[^ ^52^ ^]^ Accordingly, this Nb‐CAR.BiTE‐*γδ*T therapy likely has broad clinical applicability.

The naturally high binding capacity, solubility, stability, smaller size, and lower immunogenicity indicate the potential of Nbs in the construction of multi‐specific CARs for application to heterogeneous solid tumors.^[^ ^36^ ^]^ On the other hand, IVT mRNA allows for great engineering flexibility,^[^ ^53^ ^]^ and mRNA‐electroporated CAR‐T cell products do not cause insertional mutations and long‐term toxicity.^[^ ^54^ ^]^ In our setting, the mRNA‐driven strategy showed rapid CAR expression, superior cell expansion, and Nb‐BiTE secretion (Figure 4A–C). We also found that mRNA‐driven and lentiviral‐driven Nb‐CAR.BiTE‐*γδ*T cells exerted comparable therapeutic effects in vivo (Figure S4, Supporting Information), which is similar to the findings in a previous study.^[^ ^55^ ^]^ In addition, recent clinical studies have indicated that mRNA‐driven CAR‐T, NK, and *γδ*T cells are well‐tolerated and show promising results in patients with solid tumors.^[^ ^56^, ^57^, ^58^ ^]^ Moreover, mRNA‐based manufacturing may be more cost‐ and time‐effective based on a comparison with viral vector approaches.^[^ ^53^ ^]^ Therefore, we believe that mRNA‐driven Nb‐CAR.BiTE‐*γδ*T cells should be a potent and effective strategy with minimal side effects in vivo.

However, we still consider that lentiviral vector‐transduced Nb‐CAR.BiTE‐*αβ*T cells might have the potential for conversion into “memory Nb‐CAR.BiTE‐T cells.” These memory stem cell clones could contribute to the long‐term persistence of the circulating Nb‐CAR.BiTE‐T cell pool and exert long‐lasting tumor control.^[^ ^59^ ^]^ In the present study, we aimed to leverage the advantages of mRNA delivery and the *γδ*T platform for establishing cell therapies with natural allogeneic potential and without long‐term toxicity.^[^ ^29^, ^54^, ^60^ ^]^ Due to the monthly lifespan of *γδ*T cells^[^ ^60^ ^]^ and the natural transient expression of mRNA, we believe that repeated treatment cycles of Nb‐CAR.BiTE‐*γδ*T infusion may represent a solution for the long‐term control of solid tumors. Additionally, switching to a self‐amplifying mRNA (SAM) system might increase the persistence of Nb‐CAR.BiTE without the risk of insertional mutagenesis.^[^ ^61^ ^]^ Since TCR knockout impairs the persistence of CAR‐T cells from preclinical and clinical findings,^[^ ^62^, ^63^ ^]^ disruption of HLA molecule *β*2‐microglobulin in *αβ*T cells with stable Nb‐CAR.BiTE expression should provide for allogeneic potential and long‐lasting persistence.^[^ ^64^ ^]^ In fact, we found that HLA‐G and PD‐L1 were not downregulated in persistent tumors in mice after repeated Nb‐CAR.BiTE‐*γδ*T challenge, suggesting that antigen escape might not be the cause of treatment failure. Therefore, we hypothesize that other mechanisms are involved in immune evasion of cancer cells from our Nb‐CAR.BiTE‐*γδ*T, which will be further investigated.

Although the properties of Nbs and mRNA‐electroporation may reduce toxicity concerns, preclinical evaluations of the possible toxicity are required. In this study, there were no obvious toxicity‐related signs caused by Nb‐CAR.BiTE‐*γδ*T cells in vitro and in vivo (Figures S8 and S10, Supporting Information). The secreted Nb‐BiTE was only detectable in tumor tissues (Figure 8D,H). Moreover, our preliminary results revealed that Nb‐CAR.BiTE‐*γδ*T cells did not induce antidrug antibody (ADA) against the ecto‐Nb‐CAR and secreted‐Nb‐BiTE moieties in a mouse model (data not shown). These findings imply the multiple anti‐tumor potential of our Nb‐CAR.BiTE‐*γδ*T and the minimal safety concerns.

## 4 Conclusion 

In conclusion, we have developed a dual‐targeting mRNA‐engineered Nb‐CAR.BiTE‐V*δ*2 *γδ*T therapy is capable of overcoming the HLA‐G/PD‐L1 checkpoint dilemma and even destroying antigen‐inadequate tumor cells, which leads to potent anti‐tumor activity with no apparent toxicity. We are convinced that this therapeutic strategy has extensive potentials in future clinical practice, especially for allogeneic applications.

## 5 Experimental Section

### Reagent and Antibodies

Zoledronic acid, LPS (lipopolysaccharide), puromycin, and G418 were obtained from Sigma‐Aldrich. Antibodies against human HLA‐G (sc‐21799) and *β*‐actin (sc‐47778) were purchased from Santa Cruz Biotechnology. PD‐L1 antibody (17952‐1‐AP) and PD‐L1 (22C3) were purchased from Proteintech and Agilent Technologies, respectively. Antibodies specific for CD3*ε* (NB600‐1441), PD‐L1 (FAB1561R, FAB1561G), CD4 (FAB3791R), and CD8 (NBP2‐34590AF488) were purchased from Novus Biologicals. HLA‐G (PE: #335906, Alexa Fluor 488: #335918), PD‐1 (#367412), CD3 (#317318), CD8 (#344704), CD56 (#304611), CD66b (#305104), and V*δ*2 (#331418) were purchased from BioLegend. Anti‐Tyk2 (phospho‐Tyr1054/1055) (orb505746) antibody was obtained from Biorbyt. Antibodies specific for phospho‐ZAP70/Syk (Tyr319, Tyr352) (#MA5‐36963) and HLA‐G (MEM‐G/2: #MA1‐19394, 87G: MA1‐10356) were purchased from Thermo Fisher Scientific. Antibodies for CD14 (#12‐0149‐42), TCR*γδ* (#12‐9959‐42), TCR*αβ* (#11‐9955‐42), and NKG2D (#12‐5878‐42) were purchased from eBioscience. V*γ*9 (#555732), hMito (ab92824), and VHH (iFluor 647: A02019, HRP: A2016) were purchased from BD Pharmingen, Abcam, and GenScript, respectively. Phospho‐Stat2 (Tyr690) (#77366) and HLA‐G (#79769) were obtained from Cell Signaling Technology. Recombinant human interleukin 2 (IL‐2) was obtained from Thermo Fisher Scientific.

### Cells Lines, Primary Human Cells, and PBMC Donors

Human triple‐negative breast cancer (TNBC) cell lines MDA‐MB‐231 and MDA‐MB‐231‐Luc, human lung adenocarcinoma cell lines A549 and A549‐Luc, and human pancreatic cancer cell line AsPC‐1 were cultured in RPMI‐1640 medium. The human ovarian cancer cell line SKOV3 and human glioblastoma cell line DBTRG‐05MG were cultured in Mycoy's 5A medium and Dulbecco's modified Eagle medium, respectively. The human NK cell line NK‐92 was cultured in X‐VIVO15 medium. All media were supplemented with 10% fetal bovine serum.

Primary human cell cultures (2 donors of each tissue type) were obtained as follows: Umbilical vein endothelial cells (HUVEC) were cultured in vascular cell basal medium (ATCC) supplemented with 0.2% bovine brain extract, 5 ng mL^−1^ rhEGF, 0.75 U mL^−1^ heparin sulfate, 1 µg mL^−1^ hydrocortisone hemisuccinate, 50 µg mL^−1^ ascorbic acid, and 2% fetal bovine serum. Renal cortical epithelial cells (HRCEs) and renal mixed epithelial cells (HRECs) were cultured in renal epithelial cell basal medium (ATCC) supplemented with 10 nmol L^−1^ triiodothyronine, 10 ng mL^−1^ rhEGF, 100 ng mL^−1^ hydrocortisone hemisuccinate, 5 µg mL^−1^ insulin and transferrin, and 1 µmol L^−1^ epinephrine. Corneal epithelial cells (HCEpCs) were maintained in corneal epithelial cell basal medium (ATCC) supplemented with 5 µg mL^−1^ apo‐transferrin and insulin, 1 µmol L^−1^ epinephrine, 0.4% extract P, 100 ng mL^−1^ hydrocortisone hemisuccinate, and 0.1% CE growth factor. Thymic epithelial cells (HTyEpiCs), hepatocytes, pulmonary alveolar epithelial cells (HPAEpiCs), and pulmonary microvascular endothelial cells (HPMECs) were cultivated in thymic epithelial cell medium, hepatocyte medium, alveolar epithelial cell medium, and endothelial cell medium (ScienCell Research Laboratories), respectively. Bronchial epithelial cells (HBEpCs) were cultured in a bronchial/tracheal epithelial growth medium (Sigma‐Aldrich). Pancreatic islet cells (HP islet cells), intestinal epithelial cells (HInEpCs), and bone marrow CD34^+^ (HBM CD34^+^) cells were maintained in the complete medium supplied by Aobious, Cell Applications, and ATCC, respectively.

Peripheral blood (10–50 mL) from 12 healthy donors was collected through the Translation Cell Therapy Center (CMUH110‐REC2‐001). Donors ranged in age from 25 to 40. PBMCs were isolated from whole blood using density gradient centrifugation with Ficoll‐Paque Plus (#17144003, Cytiva). The acquisition of human peripheral blood mononuclear cell samples from donors and tissue sample studies were approved by Research Ethics committee of China Medical University & Hospital, Taichung, Taiwan (Approval number: CMUH110‐REC2‐001).

### ELISA and Competitive ELISA

The levels of IL‐17A (#A35611), IFN‐*γ* (#A35576), TNF‐*α* (#KHC3011), and granzyme b (#A44238) in the supernatants were quantified using ELISA kits (Thermo Fisher Scientific) according to the manufacturer's instructions. For competitive ELISA, each well in a 96‐well plate was coated with 0.2 µg recombinant HLA‐G (TP305216, OriGene) or PD‐L1 (#10084‐HNAH, Sino Biological) protein at 4 °C overnight. After washing with phosphate‐buffered saline and Tween 20, the wells were incubated with 0.2 µg biotinylated LILRB1(#16014‐H08H‐B, Sino Biological) or KIR2DL4 (13052‐HCCS, Sino Biological) with or without HLA‐G Nbs (10^−3^–10^−10^ mole L^−1^) for 2 h. Next, the wells were washed and blocked with 5% bovine serum albumin (BSA) containing phosphate‐buffered saline (PBS) for 1 h, followed by incubation with horseradish peroxidase (HRP)‐conjugated streptavidin for 15 min. After washing, 50 µL tetramethylbenzidine (TMB) was added to each well and the absorbance values were measured with an ELISA reader using a 450 nm channel.

### Binding Affinity of HLA‐G Nb Moiety to HLA‐G Isoforms

Full‐length HLA‐G‐*β*2M recombinant protein (containing *α*1‐*α*3 domains and a ligated *β*2M protein, which correspond to the G1 and G5 isoforms, respectively) was purchased from Creative BioMart (HLA‐G‐4305H), and the G2 and G6 isoforms (consisting of the *α*1 and *α*3 domains, aa. 1–114 and following 207–308), the G4 isoform (*α*1 and *α*2 domains, aa. 1‐206 and following 299‐308), and the G3 and G7 isoforms (*α*1 domain, aa. 1‐114 and following 299‐308) as well as HLA‐G Nb moiety‐Fc recombinant protein were manufactured by Leadgene Biomedical Inc using HEK‐293 cell‐expressing system. For the binding affinity analysis of HLA‐G Nb moiety and commercial mAbs (87G, MEM‐G/2, and 4H84), 100 µL of recombinant HLA‐G isoforms (10 µg mL^−1^) were coated on 96‐well plate at 4 °C overnight. After washing and blocking with 3% BSA‐containing PBS, each well was incubated with HLA‐G Nb moiety or HLA‐G mAbs at the indicated concentrations (0, 0.001, 0.001, 0.01, 0.1, 1, or 10 µg mL^−1^) for 2 h. Then, the wells were stained with HRP‐conjugated secondary antibody (1:5000) for 1 h. After washing, 50 µL TMB was added to each well and the absorbance at 450 nm were measured with an ELISA reader.

### PD‐L1 Blockade Bioassay of PD‐L1 Nbs

The PD‐L1 blockade activity of PD‐L1 Nbs clone#1 and #2 were determined using a PD‐1/PD‐L1 Blockade Bioassay kit (J1252, Promega) according to the manufacturer's instructions. Briefly, PD‐L1 aAPC/CHO‐K1 cells (5 × 10^4^ cells/100 µL each well) were seeded in a 96‐well plate. The next day, each well was incubated with PD‐L1 Nb clone#1, clone#2, or atezolizumab (1380723‐44‐3, Roche) at the indicated concentrations (10^−3^–10^−10^ mole L^−1^) and then co‐incubated with PD‐1 effector cells (5 × 10^4^ cells/each well). After 6 h, 80 µL of Bio‐Glo Reagent was added to each well and incubated for 5 min. Luminescence was measured by using a luminescence plate reader. Data were calculated as the average relative light units (RLUs) from the triplicate repeats. Graph data are shown as RLUs versus Log10 [antibody] mole L^−1^. The fit curves were used to determine the EC50 value of PD‐L1 Nbs and atezolizumab using SigmaPlot v14. The EC50 value of the antibody response was determined using an appropriate curve fitting software.

### Surface Plasmon Resonance (SPR) analysis for Nb Binding Affinity

Research‐grade nitrilotriacetic acid chips (BR100034, Cytiva) were used for SPR analysis with BIAcore T200 (GE Healthcare). Briefly, 20 µg mL^−1^ dilute protein (recombinant HLA‐G, PD‐L1, or CD3*ε*/*δ* (CDD‐HR2W3, ACROBiosystems)) was used in 10 mmole L^−1^ buffer solutions (pH 6.0), and maximum surface retention for immobilization on the chip was achieved by selecting surface preparation and the condition providing higher surface ligand concentrations (HLA‐G, PD‐L1, CD3*ε* Nbs) on the chip. Subsequently, regeneration scouting and surface performance tests were performed by selecting regeneration scouting and surface performance test, followed by the regeneration method to run the experiment. Subsequently, binding analysis and direct binding were selected to investigate protein binding. Finally, kinetic analysis and chose mass transfer were selected to run the kinetic assay concomitantly with the binding experiment. Data analysis was performed using the kinetic constants to determine the K_D_ values.

### Expansion of *γδ*T Cells from *αβ*T Cell‐Depleted PBMCs

Healthy donor‐derived PBMCs were depleted with *αβ*T cells by using the EasySep Human TCR Alpha/Beta Depletion Kit (#17847, STEMCELL Technologies) according to the manufacturer's instructions. Briefly, 1.2 × 10^8^ PBMCs were resuspended in 1 mL PBS containing 1% FBS, and 25 µL depletion cocktail was added. After incubation for 5 min, the cells were gently mixed with 25 µL magnetic particles and PBS was added to a volume of 4 mL. Then, the samples were immediately placed in the EasySep Magnet (#18000, STEMCELL Technologies). Three minutes later, the cell suspensions were poured into new tubes. The enrichment and expansion of *γδ*T cells from PBMCs have been previously described.^[^ ^65^ ^]^ The *αβ*T‐depleted PBMCs were cultured for 2 weeks in X‐VIVO15 medium (Lonza) containing zoledronic acid (5 µmole mL^−1^), 1000 IU mL^−1^ IL‐2, and 10% human AB serum. The final cell numbers were recorded, and their purity and phenotypic markers were determined through flow cytometry using CD27/CD45RA, CD3/NKG2D, V/V*δ*2, CD4/CD8, CD3/CD56, CD3/CD19, CD3/CD14, CD3/CD66b, and TCR*αβ*/TCR antibody panels. Finally, the *αβ*T‐depleted *γδ*T cells were subjected to subsequent experiments.

### Isolation of Primary CD3^+^ T, CD4^+^ T, CD8^+^ T, and NK cells

Human CD3, CD4, CD8, and NK cells were isolated from PBMCs using the EasySep Human CD3^+^ T (#17851), CD4^+^ T (#17 952), CD8^+^ T (#17 953), and NK Cell Isolation Kits (#17 955) (STEMCELL Technologies) according to the manufacturer's instructions. Briefly, 1 × 10^8^ PBMCs were resuspended in 2 mL PBS containing 1% FBS, and then 50 µL Isolation Cocktail was added. The samples were then incubated at 25 °C for 5 min and gently mixed with 50 µL magnetic particles, and then PBS was added to a volume of 2.5 mL. The cells were then placed into the EasySep Magnet. After incubation for 3 min, the enriched cell suspensions were poured into new tubes and their purity was examined by flow cytometry using antibodies against CD3, CD4, CD8, or CD56. Finally, the isolated cells were subjected to subsequent experiments.

### Construction of IVT mRNA and Lentiviral Vectors Encoding Nb‐CAR.BiTE

HLA‐G‐, PD‐L1‐, and CD3*ε*‐specific Nbs were generated using the Camel Naïve Single Domain Antibody Library (CaVHHL‐5, Creative Biolabs). Briefly, after four rounds of panning with recombinant HLA‐G, PD‐L1, or CD3*ε* antigen (binding/washing/elution), approximately 100 clones were selected, followed by the selection of the positive monoclonal phages by phage ELISA. Next, the positive clones were sequenced. All clones were analyzed by SPR binding assay, functional assays, and competitive ELISA, with clone#1 and #2 of HLA‐G Nb and PD‐L1 Nb clones as well as clone#2 of CD3*ε* Nb being selected. Subsequently, their sequences were used to build the Nb‐CAR.BiTE constructs that comprised CD8*α* leading peptide, HLA‐G Nb clone#1, triple repeated GGGGS linker and HLA‐G Nb #2, followed by the CD8 hinge/transmembrane domain (amino acids 144‐203), 4‐1BB cytosolic domain (amino acids 214‐255) inserted with a Tyk‐binding motif from IFNAR1 (amino acids 458–520) at amino acid 230, and CD3*ξ* cytosolic domain (amino acids 52–164) inserted with an integrated ITAM from DAP‐12 (amino acids 62‐64 + 80‐108) at amino acid 159. This was followed by fusion with the full‐length self‐cleavage peptide P2A. Subsequently, IL‐2 leading peptide following PD‐L1 Nb clone #1, triple repeated GGGGS linker and PD‐L1 Nb clone #2 were ligated with triple repeats of GGGGS linker and subsequently linked with the CD3*ε* Nb of clone #2. This construct was processed with codon optimization (GenSmart Codon Optimization tool), and gene synthesized by GenBrick synthesis service, and then cloned into pcDNA3.1 (Plasmid #138209, Addgene) with the T7 promoter, 5’UTR and 3’UTR for IVT mRNA production and into pCAR (Puroless) lentiviral vector (Creative Biolabs) using the EF‐1*α* promoter at the EcoRI/XbaI cloning site. The used sequences for constructing Nb‐CAR.BiTE are listed in **Table** **1**.

**Table 1:** Gene sequences for engineering Nb‐CAR.BiTE construct (N.A.: non‐available, untranslated sequence)

Gene	DNA sequence	Amino acid sequence
CD3*ε* Nb#2	CATGTGCAGCTGGTGGAGTCTGGGGGAGGCTCGGTGCAGGCTGGGGGGTCTCTGAGACTCTCCTGTACAGTGTCTGGAGTCATCTTTAAGAACGAGTACATGGGCTGGTTCCGCCAGGCCCCAGGGAAGGAGCGCGAGGGGGTCGCAGCAGCTTCGCCTGGTGGAACGATTACATACTATGGGGACTCCGTGAAGGGCCGATTCACCATCTCCCGAGACAATGCCAAGAACACGGTGTATCTGCAAATGAACCGCCTGAAACCTGAGGACACTGCCATGTACTACTGTGCGTTGGATCCCTCGACTACGTCATGGTCTATCATCCGCCACGGTCCATCGCTTTGGCGTTATAGCGGCCGGGGGACCCAGGTCACCGTCTCCTCA	HVQLVESGGGSVQAGGSLRLSCTVSGVIFKNEYMGWFRQAPGKEREGVAAASPGGTITYYGDSVKGRFTISRDNAKNTVYLQMNRLKPEDTAMYYCALDPSTTSWSIIRHGPSLWRYSGRGTQVTVSS
CD3*ξ* (intracellular domain)	AGAGTGAAGTTCAGCAGGAGCGCAGACGCCCCCGCGTACCAGCAGGGCCAGAACCAGCTCTATAACGAGCTCAATCTAGGACGAAGAGAGGAGTACGATGTTTTGGACAAGAGACGTGGCCGGGACCCTGAGATGGGGGGAAAGCCGCAGAGAAGGAAGAACCCTCAGGAAGGCCTGTACAATGAACTGCAGAAAGATAAGATGGCGGAGGCCTACAGTGAGATTGGGATGAAAGGCGAGCGCCGGAGGGGCAAGGGGCACGATGGCCTTTACCAGGGTCTCAGTACAGCCACCAAGGACACCTACGACGCCCTTCACATGCAGGCCCTGCCCCCTCGC	RVKFSRSADAPAYQQGQNQLYNELNLGRREEYDVLDKRRGRDPEMGGKPQRRKNPQEGLYNELQKDKMAEAYSEIGMKGERRRGKGHDGLYQGLSTATKDTYDALHMQALPPR
CD8*α* leading peptide	ATGGCCCTCCCTGTCACCGCCCTGCTGCTTCCGCTGGCTCTTCTGCTCCACGCCGCTCGGCCC	MALPVTALLLPLALLLHAARP
CD8*α* (hinge/transmembrane domain)	ACCACGACGCCAGCGCCGCGACCACCAACACCGGCGCCCACCATCGCGTCGCAGCCCCTGTCCCTGCGCCCAGAGGCGTGCCGGCCAGCGGCGGGGGGCGCAGTGCACACGAGGGGGCTGGACTTCGCCTGTGATATCTACATTTGGGCCCCTCTGGCTGGTACTTGCGGGGTCCTGCTGCTTTCACTCGTGATCACTCTTTACTGT	TTTPAPRPPTPAPTIASQPLSLRPEACRPAAGGAVHTRGLDFACDIYIWAPLAGTCGVLLLSLVITLYC
DAP‐12 ITAM	TACTTCCTGCGGAAACAGCGTATCACTGAGACCGAGTCGCCTTATCAGGAGCTCCAGGGTCAGAGGTCGGATGTCTACAGCGACCTCAACACACAG	YFLRKQRITETESPYQELQGQRSDVYSDLNTQ
HLA‐G Nb#1	GAGGTGCAGCTGGTGGAGTCTGGGGGAGGCTCGGTGCAGGCTGGAGGGTCTCTGAGACTCTCCTGTGATGCCTCTAAATACACCTACTTTAGGAACTGCATGGGCTGGTTCCGCCAGGTTCCTGGGGCGGAGCGCGAGGGGGTCGCAACTATTGATAGTGCTGGTGGCACCGAGCTACGCAGATTTTGTGAAGGGCCGATTCACCATCTCCCGAGACAACGCCAAGACTGCTCTGTATCTGCAAATGAACAGCCTGAAACCTGAGGACACTGCCATGTACTACTGTTTCGGTGGTAGCTGGTACAAAGGCAGCTGCATATATGAGTATAACTACTGGGGCCAGGGGACCCAGGTCACCGTCTCCTCA	EVQLVESGGGSVQAGGSLRLSCDASKYTYFRNCMGWFRQVPGAEREGVATIDSAGGTSYADFVKGRFTISRDNAKTALYLQMNSLKPEDTAMYYCFGGSWYKGSCIYEYNYWGQGTQVTVSS
HLA‐G Nb#2	CATGTGCAGCTGGTGGAGTCTGGGGGAGGCTCGGTGCAGGCTGGAGGGTCTCTGAAACTCTCCTGTGTAACCTCTGCATATACCTTCAGCGCCAGTGGCAATTGCATGGGCTGGCTTCGCCAGGCTCCAGGGAAGGGGCGCGAGGGAATCGCGGCTACATATACGAGAAGTGCTAAGACATACTATGCCGACTCGGTGAAGGGGCGATTCACCATCTCCCAAGACAACGCCAAGAACACGGTGTATCTACAAATGAACGGCCTGAAACCTGAGGACACTGCTACGTATTACTGTGCGGTGGCCCGCTGTGCTGGGCGGCCCGATCGCTCAACCCTCACTTCCTTTGCATGGTGGGGCCAGGGGACGCAGGTCACCGTCTCCTCA	HVQLVESGGGSVQAGGSLKLSCVTSAYTFSASGNCMGWLRQAPGKGREGIAATYTRSAKTYYADSVKGRFTISQDNAKNTVYLQMNGLKPEDTATYYCAVARCAGRPDRSTLTSFAWWGQGTQVTVSS
IFNAR1 (truncated)	AAAGTCTTCTTGAGATGCATCAATTATGTCTTCTTTCCATCACTTAAACCTTCTTCCAGTATAGATGAGTATTTCTCTGAACAGCCATTGAAGAATCTTCTGCTTTCAACTTCTGAGGAACAAATCGAA*AGA*TGTTTCATAATTGAAAATATAAGCACAATTGCTACAGTAGAAGAAACTAATCAAACTGATGAAGATCATAAAAAATACAGTTCCCAAACTAGCCAAGATTCAGGAAATTATTCTAATGAAGATGAAAGCGAAAGTAAAACAAGTGAAGAACTACAGCTGGTCTTGGACTCC	KVFLRCINYVFFPSLKPSSSIDEYFSEQPLKNLLLSTSEEQIERCFIIENISTIATVEETNQTDEDHKKYSSQTSQDSGNYSNEDESESKTSEELQLVLDS
IL‐2 leading peptide	ATGTACAGGATGCAACTCCTGTCTTGCATTGCACTAAGTCTTGCACTTGTCACAAACAGT	MYRMQLLSCIALSLALVTNS
Kozak sequence	GCCACC	N.A.
PD‐L1 Nb#1	CATGTGCAGCTGGTGGAGTCTGGGGGAGGCTTGGTGCAGCCTGGGGGGTCTCTGAGACTCTCCTGTGCAGCCTCTGGATTCACCTTCAGTAGCAAGGCCATGAGCTGGGTCCGCCAGGCTCCAGGGAAGGGACTCGACTGGGTCTCAACCATTAATAGTGGTGGTGGTAACACATACTATTCAGACTCCGTGAAGGGCCGATTCACCATCTCCAGAGACAACGCCAAGAACACGCTGTATCTGCAATTGAACAGCCTGAAAACTGAGGACACGGCCATGTATTACTGTTCCCGTTGTAGCGATATTTACTGCGGAGGGCAATATACGTATCGGGGCCAGGGGACCCTGGTCACTGTCTCCTCA	>HVQLVESGGGLVQPGGSLRLSCAASGFTFSSKAMSWVRQAPGKGLDWVSTINSGGGNTYYSDSVKGRFTISRDNAKNTLYLQLNSLKTEDTAMYYCSRCSDIYCGGQYTYRGQGTLVTVSS
PD‐L1 Nb#2	GAGGTGCAGCTGGTGGAGTCTGGGGGAGGCTTGGTGCAGCCTGGGGGGTCTCTGAGACTCTCCTGTGTAGCCTCTGGATTCACCTTCAGTAGCATTGGCATGAGTTGGGTCCGCCAGGCTCCAGGGAAGGGGCTCGAGTGGGTCTCAGGTCTGAATCCTGTTGGTAGTCACACAGGCTATGCAGACTCCGTAAAGGGCCGATTCACCATCTCCAGAGACAACGCCAAGAATACGCTGCATCTGCAGTTGAACAGCCTGAAAACTGAGGACACGGCCATGTATTACTGTCAAAGAGGTTATACTTGTAGCGGTGATTTGTGCGAAAGGGGTCAGGGGACCCAGGTCACTGTCTCCTCA	EVQLVESGGGLVQPGGSLRLSCVASGFTFSSIGMSWVRQAPGKGLEWVSGLNPVGSHTGYADSVKGRFTISRDNAKNTLHLQLNSLKTEDTAMYYCQRGYTCSGDLCERGQGTQVTVSS
P2A	GCCACAAATTTCAGCCTGCTGAAACAGGCCGGCGACGTGGAAGAGAACCCTGGACCT	ATNFSLLKQAGDVEENPGP
Triple repeats of GGGGS linker	GGAGGCGGAGGTTCTGGAGGCGGAGGTTCTGGAGGCGGAGGTTCT	GGGGSGGGGSGGGGS
Tyk2‐binding motif (IFNAR1)	AAAGTCTTCTTGAGATGCATCAATTATGTCTTCTTTCCATCACTTAAACCTTCTTCCAGTATAGATGAGTATTTCTCTGAACAGCCATTGAAGAATCTTCTGCTTTCAACTTCTGAGGAACAAATCGAAAGATGTTTCATAATTGAAAATATAAGCACAATTGCTACAGTAGAAGAAACTAATCAAACT	KVFLRCINYVFFPSLKPSSSIDEYFSEQPLKNLLLSTSEEQIERCFIIENISTIATVEETNQT
T7 promoter	TAATACGACTCACTATAGGG	N.A.
3’‐UTR (*α*‐globin)	GCTGGAGCCTCGGTAGCCGTTCCTCCTGCCCGCTGGGCCTCCCAACGGGCCCTCCTCCCCTCCTTGCACC	N.A.
4‐1BB (intracellular domain)	ATCATCTCCTTCTTTCTTGCGCTGACGTCGACTGCGTTGCTCTTCCTGCTGTTCTTCCTCACGCTCCGTTTCTCTGTTGTTAAACGGGGCAGAAAGAAACTCCTGTATATATTCAAACAACCATTTATGAGACCAGTACAAACTACTCAAGAGGAAGATGGCTGTAGCTGCCGATTTCCAGAAGAAGAAGAAGGAGGATGTGAACTG	IISFFLALTSTALLFLLFFLTLRFSVVKRGRKKLLYIFKQPFMRPVQTTQEEDGCSCRFPEEEEGGCEL
4‐1BB (TRIF binding motif)	CCAGTACAAACTACTCAAGAGGAAGATGGCTGTAGCTGCCGATTTCCAGAAGAAGAAGAAGGAGGATGTGAACTG	PVQTTQEEDGCSCRFPEEEEGGCEL
5’‐UTR (hemoglobin subunit beta)	ACATTTGCTTCTGACACAACTGTGTTCACTAGCAACCTCAAACAGACACC	N.A.

### Nb‐CAR and Nb‐CAR.BiTE mRNA Synthesis

Codon‐optimized mRNA for Nb‐CAR and Nb‐CAR.BiTE mRNA were manufactured by MISSION BIOTECH CO., LTD. The in vitro transcription of Nb‐CAR.BiTE IVT mRNA was performed by using HiScribe T7 ARCA (anti‐reverse Cap Analog 3′‐O‐Me‐m7G(5′)ppp(5′)G) mRNA Kit (with tailing) (E2060, New England Biolabs) according to the manufacturer's instructions. Briefly, pcDNA3.1 vectors encoding Nb‐CAR.BiTE were incubated EcoRI (NEB #R3101, New England Biolabs) and XbaI (NEB #R0145, New England Biolabs) for 30 min at 37 °C, then the linearized Nb‐CAR.BiTE template DNA was isolated by agarose gel electrophoresis and phenol chloroform extraction. Then the linear DNA templates were mixed with 2× ARCA/NTP Mix, T7 RNA polymerase Mix, and RNase inhibitor and T7 RNA polymerase for 45 min incaution at 37 °C. The DNA template was removed by adding DNase and incubation for 15 min at 37 °C. Then processed with poly(A) tailing reaction for 30 min at 37 °C. The quality of the synthesized mRNA was determined by agarose gel electrophoresis and Sanger sequencing of cDNA.

### Nb‐BiTE Recombinant Protein Production

Codon‐optimized pcDNA3.1‐encoded Nb‐BiTE for recombinant protein (IL‐2 leading peptide following PD‐L1 Nb #1, triple repeated GGGGS linker, and PD‐L1 Nb#2 were ligated with triple repeats of GGGGS linker and subsequently fused with the CD3*ε* Nb#2) was manufactured by Leadgene Biomedical Inc. using an HEK‐293T expressing system. Briefly, the pCMV vectors encoding Nb‐BiTE were transfected by Lipofectamine 3000 according to the manufacturer's instructions. The secreted Nb‐BiTE was harvested and concentrated by a vacuum centrifugal concentrator, then the concentrated supernatants were loaded onto a gravity‐flow column containing 1 mL of prepacked resin and incubated at room temperature for 30 min. Before elution of the Nb‐BiTE by addition of elution buffer containing 300 × 10^‐3^ m imidazole, the column was washed twice with increasing imidazole concentrations of 20 × 10^‐3^ and 40 × 10^‐3^ m. Removal of imidazole and buffer exchange was achieved by dialysis against PBS using a cellulose ester membrane with a molecular weight cut‐off of 3.5–5 kDa (Spectrum Laboratories). The quality of the purified Nb‐BiTE was determined by SDS‐PAGE electrophoresis and SPR assay.

Electroporation of IVT mRNA to *γδ*T cells and determination of Nb‐CAR expression through fluorescence‐activated cell sorting analysis: Briefly, 1 × 10^8^ *αβ*T cell‐depleted *γδ*T cell products were electroporated with 200 µg of Nb‐CAR or Nb‐CAR.BiTE IVT mRNA by 4D‐Nucleofector LV Unit (Lonza) using the program code as primary P3 cells/CM137 according to the manufacturer's instructions. To determine Nb‐CAR expression, 1 × 10^6^ Nb‐CAR or Nb‐CAR.BiTE *γδ*T cells were harvested at the indicated time points and then stained with iFluor 647‐conjugated anti‐VHH antibody or isotype control (#51‐4616‐82, eBioscience). After washing twice using 1% BSA containing PBS, Nb‐CAR expression was determined by flow cytometry, with the isotype antibody‐staining cells as the background controls. One day after electroporation, the Nb‐CAR or Nb‐CAR.BiTE *γδ*T cells were subjected to subsequent experiments.

### Evaluation of the Effects of Nb‐BiTE Secreted from Nb‐CAR‐*γδ*T Cells

Cell‐impenetrable transwell membranes (0.4 µm pore size) were used to evaluate the effects of the released Nb‐BiTE, as previously reported.^[^ ^30^ ^]^ Briefly, 1 × 10^5^ target tumor cells were seeded on 12‐well plates. After 30 min, the effector cells (CD4, CD8, NK, parental, or Nb‐CAR‐*γδ*T cells) were added to the indicated wells at an effector (E) to target (T) ratio (E:T ratio) of 1:1, followed by exposure with or without the top well containing the cell‐impenetrable membrane and 5 × 10^5^ Nb‐CAR.BiTE‐*γδ*T, parental, or Nb‐CAR‐*γδ*T cells. At the indicated time points, the bottom cells were subjected to a LIVE/DEAD cell‐mediated cytotoxicity assay or stained with anti‐VHH antibodies, followed by analysis using flow cytometry. The effect of Nb‐BiTE on immune cells was evaluated by culturing 1 × 10^6^ CD3^+^, *γδ*T, or Nb‐CAR‐*γδ*T cells at the bottom of 12‐well plates and then exposing the cells to 1 × 10^6^ parental, Nb‐CAR‐*γδ*T, or Nb‐CAR.BiTE‐*γδ*T cells at the top well. At the indicated time points, the cell numbers were counted, and the cells of the control group were normalized as 1‐fold.

### Generation of Stable Clones with Overexpression or Silencing of HLA‐G or PD‐L1

To generate HLA‐G‐ and PD‐L1‐overexpressing cells, human pCMV1 plasmids encoding human PD‐L1 open reading frame (RC213071, Origene) were transfected into A549 cells using Lipofectamine 3000 (L3000015, Invitrogen). To obtain stable HLA‐G and PD‐L1 knockdown cells, the HLA‐G (sc‐42920‐V) or PD‐L1 (sc‐39699‐V) shRNA lentiviral particles (Santa Cruz) were transfected into H1975 and MDA‐MB‐231 cells using polybrene (8 µg mL^−1^). Subsequently, stable single‐cell clones were selected using G418 (200–400 µg mL^−1^) or puromycin (10–20 µg mL^−1^) for ≥14 d, followed by the selection of single clone‐derived sub‐cell lines based on the membrane‐bound and/or total expression levels of HLA‐G or PD‐L1 determined through flow cytometry and immunoblotting, respectively.

### LIVE/DEAD Cell‐Mediated Cytotoxicity Assay

Cytotoxicity levels in the parental, Nb‐CAR, and Nb‐CAR.BiTE‐*γδ*T and the CD4, CD8, NK, and PBMC cells were determined using the Live/Dead cell‐mediated cytotoxicity assay kit (L7010, Thermo Fisher Scientific) according to the manufacturer's instructions. Briefly, tumor cells were pre‐stained using green‐fluorescent DiOC_18_ for 15 min. After washing, the target tumor cells were cocultured with or without the indicated ratios of effector cells for 48 or 72 h, followed by staining with red‐fluorescent propidium iodide (2 µg mL^−1^). Specific lysis of target cells was determined by the percentage of killed cells (green‐fluorescent^+^/red‐fluorescent^+^) through flow cytometry analysis and normalized to the untreated control.

### Determine the Affinity of HLA‐G Nb Moiety to HLA‐G Isoforms and Quantification of Secreted Nb‐BiTE from Nb‐CAR‐*γδ*T Cells

To determine the cross‐reactive activity of HLA‐G Nb#1 and #2 and the HLA‐G Nb moiety (HLA‐G Nb#1 and #2) to HLA‐G isoforms, 100 µL recombinant HLA‐G‐*β*2M, HLA‐G *α*1*α*3, *α*1*α*2, and/or *α*1 domain proteins (10 µg mL^−1^ in PBS) were coated on 96‐well plates at 4 °C for overnight. After washing and blocking, 50 µL HLA‐G Nb moiety, HLA‐G mAb clone 87G, MEMG/2, or 4H84 (10 µg mL^−1^ in PBS) was added to each well and incubated for 2 h, The wells were washing and stained with HRP‐conjugated anti‐VHH, anti‐mouse, or anti‐rabbit secondary antibody (1:5000) for 2 h. For measuring the amounts of secreted Nb‐BiTE, 50 µL PD‐L1 recombinant protein (10 µg mL^−1^ in PBS) was added to 96‐well ELISA plates for 2 h. After blocking, the supernatants of *γδ*T cells or the mouse tissue extracts were added and incubated overnight at 4 °C. After washing, the wells were stained with HRP‐conjugated anti‐VHH secondary antibody (1:5000) for 2 h. Next, 50 µL TMB was added to each well, and the absorbance was measured by the ELISA reader at 450 nm. Recombinant PD‐L1 Nb‐BiTE was used as a standard for calculating the amounts of Nb‐BiTE in each sample.

### Immunoblotting

Briefly, each protein sample (50 µg) was subjected to sodium dodecyl sulfate‐polyacrylamide gels after electrophoresis, followed by electroblotting onto polyvinylidene difluoride membranes (IPVH00010, Millipore) on ice. After blocking and incubation with primary antibodies at 4°C overnight, the membranes were stained with secondary antibodies for 2 h at room temperature. After washing, the blots were incubated with the enhanced chemiluminescence reagent (Millipore) and analyzed using ChemiDoc Imaging Systems (Bio‐Rad Hercules).

### Mice

Six to eight week old NSG‐comparable (female, 20–22 g) and HLA‐G transgenic mice (CBA/Ca‐Tg(H2‐K‐HLA‐G,B2M)1Alm/CmwJ) (male and female, 22–25 g) for all experiments. NSG‐comparable and HLA‐G transgenic mice were originally purchased from the National Laboratory Animal Center/BioLASCO Taiwan Co. Ltd. and The Jackson Laboratory, respectively. All mice were maintained at the same animal facility at the China Medical University Hospital. All experimental mice were housed in specific pathogen‐free conditions and handled according to animal care guidelines from the Committee for Animal Experiments, China Medical University Hospital, which approved all the animal protocols (CMUIACUC‐2021‐045).

### PBMC‐Humanized Cell Line‐Derived NSG (PBMC‐CDX‐NSG) Xenograft Mouse Models

Regarding the orthotopic TNBC tumor model, MDA‐MB‐231‐luc cells (1 × 10^6^ in 100 µL PBS) were subcutaneously injected into female NSG mice at the left 4^th^ mammary gland. For constructing the orthotopic lung tumor model (*n* = 5), PD‐L1 overexpressing A549‐luc cells were resuspended in Matrigel (1 × 10^6^ cells/20 µL), followed by rapid percutaneous injection into the upper margin of the sixth intercostal rib on the right anterior axillary at a 5 mm depth. After 7 d, the mice were transplanted with PBMCs (5 × 10^6^ in 100 µL PBS). Three days later, the mice were treated with or without parental or Nb‐CAR.BiTE‐*γδ*T cells (1 × 10^7^ in 100 µL PBS) once a week for 4 weeks. Furthermore, all mice were supplemented with hIL‐2 10 000 IU per mouse and 4 µg kg^−1^ of zoledronic acid three times per week. Tumor growth was monitored by IVIS Spectrum In Vivo Imaging System (PerkinElmer) using the bioluminescent channel. The survival rates were determined with death or the MDA‐MB‐231 tumor reaching >1000 mm^3^.

### Biodistribution of *γδ*T Cells in Orthotopic PBMC‐CDX‐NSG Models

At 14 d after orthotopic inoculation of 1 × 10^6^ PD‐L1‐overexpressing A549‐luc or 3 × 10^6^ MDA‐MB‐231‐luc cells into female NSG mice (*n* = 5). Healthy donor‐derived parental, Nb‐CAR, and Nb‐CAR.BiTE‐*γδ*T cells were pre‐stained with 2 µmole L^−1^ XenoLight DiR (PerkinElmer) at 37 °C for 15 min. After washing with PBS twice, the *γδ*T cells were resuspended in PBS (1 × 10^7^ cells/100 µL) and then injected into the tumor‐bearing mice through the tail vein. At the indicated time points, the in vivo distribution of infused *γδ*T cells was detected by the IVIS system using fluorescence filters set at 710 nm excitation and 760 nm emission, while the tumor burdens were monitored using the bioluminescent channel.

### Immunohistochemistry (IHC) and Chromogenic 2‐Plexed IHC Analysis

Each section was continuously sliced to a 3 µm thickness and processed with antigen retrieval, followed by incubation with H_2_O_2_ for 20 min after washing, and then soaking with 5% BSA containing PBS. For regular IHC staining, slides were incubated with primary antibodies at 4 °C overnight. For chromogenic 2‐plexed IHC staining, HLA‐G antibody (4H84) was reacted with Discovery Teal‐HRP (#760‐247, Roche) according to the manufacturer's instructions, then the specimens were stained with Discovery Teal‐HRP‐labeled HLA‐G antibody and PD‐L1 antibody (22C3, Dako) at 4 °C overnight. After washing, the sections were stained using biotin‐conjugated secondary antibodies. Finally, the sections with polymer were stained for 10 min at room temperature, followed by incubation with DAB. Subsequently, the sections were lightly stained with hematoxylin/eosin and fixed. Positive staining cells were quantified using the CaseViewer software (ver. 2.3) (3DHISTECH Ltd.) or using HistoQuest software (ver. 7.1) (TissueGnostics) at 400 × magnification and recorded.

### Toxicity Evaluation in HLA‐G Transgenic Mice

Male and female HLA‐G transgenic mice (*n* = 5 of each gender) were injected weekly with Nb‐CAR.BiTE‐*γδ*T (1 × 10^7^ in 100 µL PBS) via tail vein for 4 weeks. The body weight, food intake, activity, diarrhea, and the MGS scores.^[^ ^66^ ^]^ were monitored twice a week until the indicated day (49 d after the first infusion of *γδ*T). Mice were sacrificed, and then their serum, brain, thymus, lung, heart, liver, stomach, intestine, colon, kidney, spleen, pancreas, bone, muscle, lymph node, eyes, and ovary or testis were harvested. The H score of HLA‐G expression and the amounts of TUNEL‐positive or human mitochondria (hMito)‐positive cells were determined in each high power field (HPF) and analyzed by the pathologist Dr. CI Jan.

### Statistical Analyses

All experiments were performed at least three independent experiments. Data were analyzed by Student's *t‐*test, paired Student's t‐test and ANOVA test using SigmaPlot 14.0 or Prism 9.0.0. Statistical differences in variables such as cell growth and cell killing, and cytokine assay data were assessed using Student's *t*‐tests and paired Student's t‐tests. Survival and tumor growth were analyzed using the Kaplan–Meier method and the log‐rank test. Statistical significance was set at a *p* value of 0.05.

## Conflict of Interest

The authors declare no conflict of interest.

## Author Contributions

S.W.H., C.M.P., S.C.C., and D.Y.C. contributed equally to this work. S.W.H., S.C.C., and D.Y.C. designed the study; S.W.H., Y.C.L., C.Y.L., P.Y.L., W.H.H., Y.T.C., W.C.T., Y.C., C.M.P., F.Y.L., and S.T.W. conducted the experiments, S.W.H., C.M.P., Y.C.L., C.C.W., Y.H.C., T.H.L., and S.C.C. acquired data; S.W.H., Y.C.L., S.T.W., and C.I.J. analyzed data; S.W.H., M.C.C., S.C.C., and D.Y.C. wrote the manuscript.

## Supporting information

Supporting Information

## Data Availability

The data that support the findings of this study are available from the corresponding author upon reasonable request.
