# Supplementary material for: BiTE‐Secreting CAR‐γδT as a Dual Targeting Strategy for the Treatment of Solid Tumors
Source: Adv Sci (Weinh). 2023 Apr 20;10(17):2206856. doi: 10.1002/advs.202206856 (PMC10265101; doi:10.1002/advs.202206856)
Supplement: Supplementary file 1 — Supporting Information [file ADVS-10-2206856-s001.pdf]

ADVANCED SCIENCE
Open Access

Supporting Information

for *Adv. Sci.*, DOI 10.1002/advs.202206856

BiTE-Secreting CAR-$\gamma\delta$T as a Dual Targeting Strategy for the Treatment of Solid Tumors

Shi-Wei Huang, Chih-Ming Pan, Yu-Chuan Lin, Mei-Chih Chen, Yeh Chen, Chia-Ing Jan, Chung-Chun Wu, Fang-Yu Lin, Sin-Ting Wang, Chen-Yu Lin, Pei-Ying Lin, Wei-Hsaing Huang, Yu-Ting Chiang, Wan-Chen Tsai, Ya-Hsu Chiu, Ting-Hsun Lin, Shao-Chih Chiu and Der-Yang Cho**

Supplementary Information

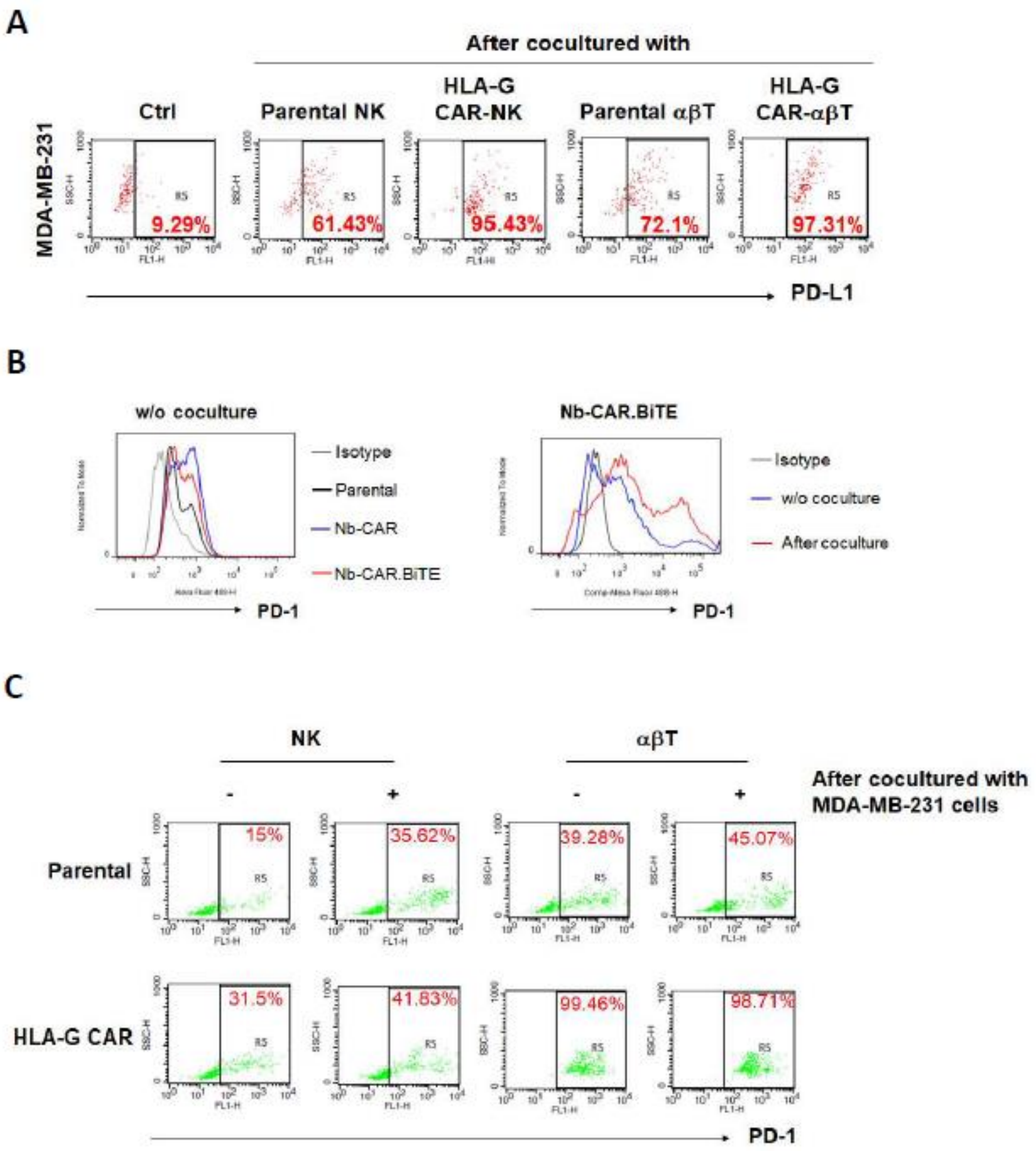

Supplementary Figure 1. PD-L1/PD-1 axis is upregulated when tumor cells engage with various effector cells carrying HLA-G CARs. A) PD-L1 was amplified in tumor cells after challenge with Nb-CAR-expressing effector cells. MDA-MB-231 cells were cocultured with parental or Nb-CAR-αβT (or NK) cells at an E:T ratio of 1:1 for 48 h and then stained with PD-L1 antibody and analyzed by flow cytometry. B,C) Significant upregulation of PD-1 in various effector cells after engagement with tumor cells. B) PD-1 expression in γδT cells after electroporation with Nb-CAR or

Nb-CAR.BiTE IVT mRNA (left panel), and PD-1 expression in Nb-CAR.BiTE-γδT cells after coculture with A549 cells (E:T = 3:1) for 48 h (right panel). C) αβT or NK cells were engineered with or without Nb-CAR and then cocultured with/without MDA-MB-231 cells (E:T = 1:1) for 48 h. PD-1 levels in these effector cells were then analyzed by flow cytometry.

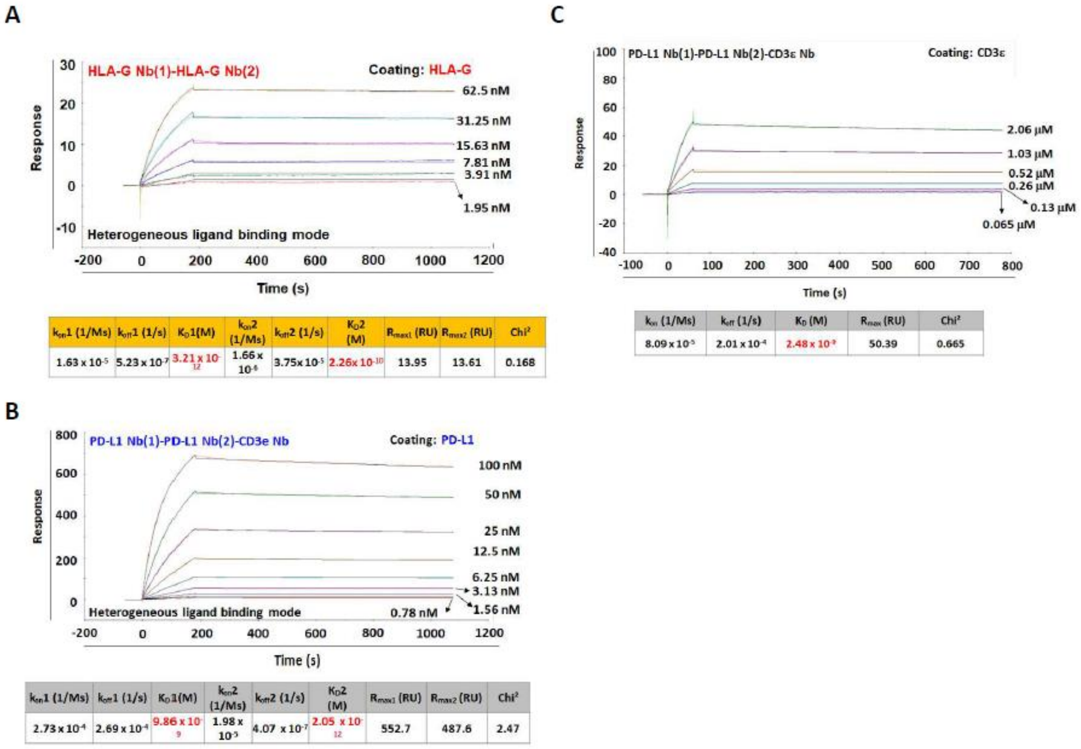

A)

$k_{on}1$ (1/Ms)	$k_{off}1$ (1/s)	K_D1(M)	$k_{on}2$ (1/Ms)	$k_{off}2$ (1/s)	K_D2 (M)	R_{max1} (RU)	R_{max2} (RU)	Chi²
1.63×10^{-5}	5.23×10^{-7}	3.21×10^{-12}	1.66×10^{-6}	3.75×10^{-5}	2.26×10^{-10}	13.95	13.61	0.168

B)

$k_{on}1$ (1/Ms)	$k_{off}1$ (1/s)	K_D1(M)	$k_{on}2$ (1/Ms)	$k_{off}2$ (1/s)	K_D2 (M)	R_{max1} (RU)	R_{max2} (RU)	Chi²
2.73×10^{-4}	2.69×10^{-4}	9.86×10^{-9}	1.98×10^{-5}	4.07×10^{-7}	2.05×10^{-12}	552.7	487.6	2.47

C)

k_{on} (1/Ms)	k_{off} (1/s)	K_D (M)	R_{max} (RU)	Chi²
8.09×10^{-5}	2.01×10^{-4}	2.48×10^{-9}	50.39	0.665

Supplementary Figure 2. Binding affinities of Nb-CAR and Nb-BiTE moieties, respectively, to their antigens. A-C) High target affinity of Nb-CAR and Nb-BiTE moieties. The binding affinities of A) HLA-G-specific Nb to recombinant HLA-G and PD-L1/CD3-targeted Nb-BiTE moiety to B) recombinant PD-L1 or C) CD3ε were measured by an SPR binding assay using heterogenous ligand binding mode analysis.

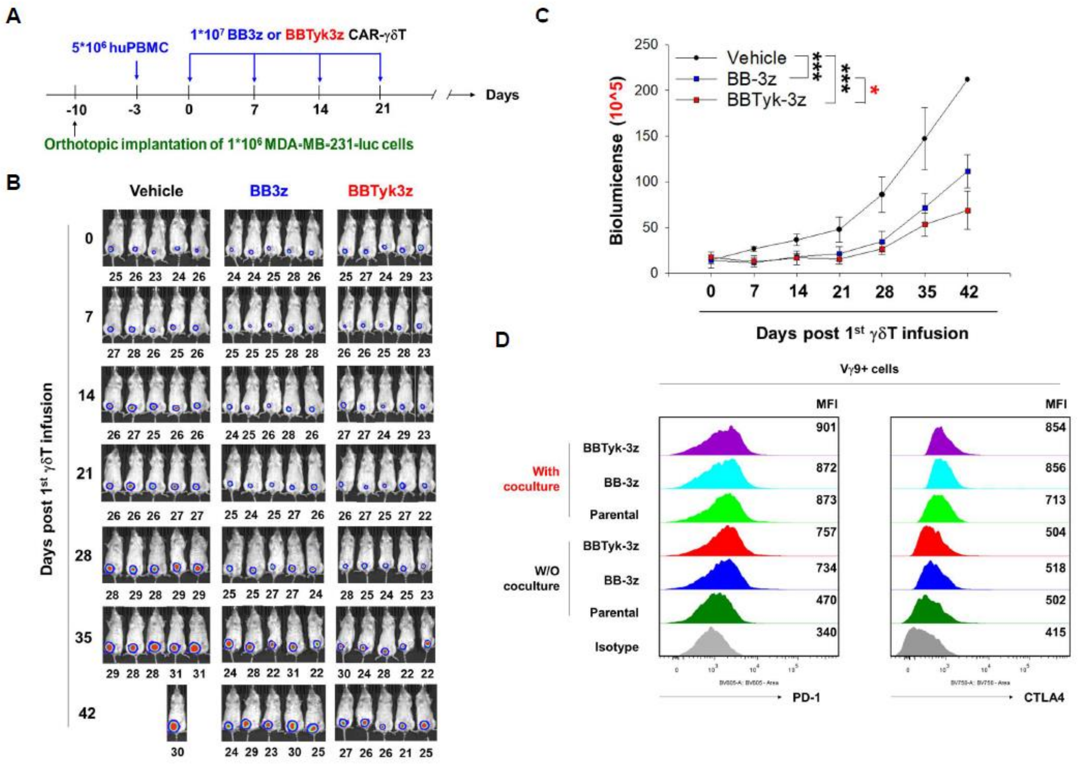

Supplementary Figure 3. Modified BBTyk-3z-based CAR ICD induced superior anti-tumor activity *in vivo*. A) Protocol for evaluating the anti-tumor activity of lentiviral-driven BB-3z- or BBTyk-3z-based Nb-CAR-γδT cells in PBMC-CDX-NSG mice bearing orthotopic breast tumors (n = 5). Seven days after inoculation with 1×10^6 MDA-MB-231-luc cells, the mice received tail vein injections of PBMCs (5×10^6 cells/mouse). Three days later, the parental, BB-3z-based, or BBTyk-3z-based Nb-CAR-γδT cells (1×10^7 cells/mouse) were injected through the tail vein once a week for 4 weeks. B,C) Superior anti-tumor activity was observed for BBTyk-3z-based Nb-CAR-γδT cells relative to BB-3z-based Nb-CAR-γδT cells in the PBMC-CDX-NSG mouse model. The tumor growth of the orthotopically inoculated MDA-MB-231 was monitored weekly through IVIS. D) BBTyk-3z CAR ICD did not show enhanced exhaustion in γδT cells compared with that of the BB-3z-based construct. Parental, BB-3z-based, or BBTyk-3z-based Nb-CAR-γδT cells were cocultured with or without MDA-MB-231 cells at an E:T of 3:1 for 72 h, and then the

expression of PD-1 and CTLA-4 on γδT cells was determined by flow cytometry. Data represent the mean ± SD; *$p < 0.05$; **$p < 0.01$; and ***$p < 0.001$ based on the Kaplan–Meier method and log-rank test.

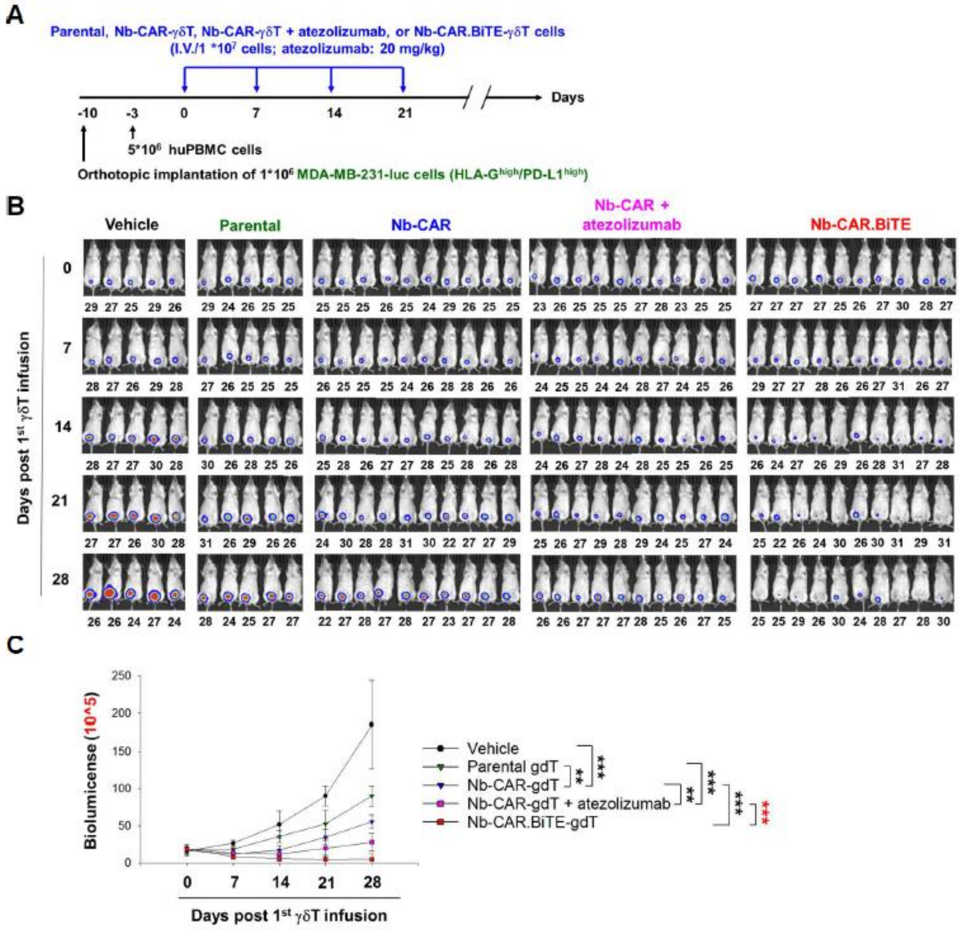

Supplementary Figure 4. Comparable anti-tumor activity of lentiviral and mRNA-driven Nb-CAR.BiTE-γδT cells *in vivo*. A) Protocol for evaluating the anti-tumor activity of lentiviral and IVT mRNA-driven Nb-CAR.BiTE-γδT cells in the PBMC-CDX-NSG mice bearing orthotopic MDA-MB-231 tumor (n = 6). Seven days after inoculation with 1 × 10^6 MDA-MB-231-luc cells, the mice received tail vein injection with PBMCs (5 × 10^6 cells/mouse). Three days later, the parental γδT, lentiviral Nb-CAR.BiTE vector-transduced (MOI = 3) or Nb-CAR.BiTE IVT mRNA electroporated γδT cells (1 × 10^7 cells/mouse) were injected through tail vein once a

week for 4 weeks. B,C) Similar anti-tumor activity between lentiviral and IVT mRNA-driven Nb-CAR.BiTE-γδT cells in the PBMC-CDX-NSG mouse model. The tumour growth of the orthotopically inoculated MDA-MB-231 were monitored weekly through IVIS. Data represent the mean ± SD; **p* < 0.05; ***p* < 0.01; and ****p* < 0.001 based on the Kaplan–Meier method and log-rank test.

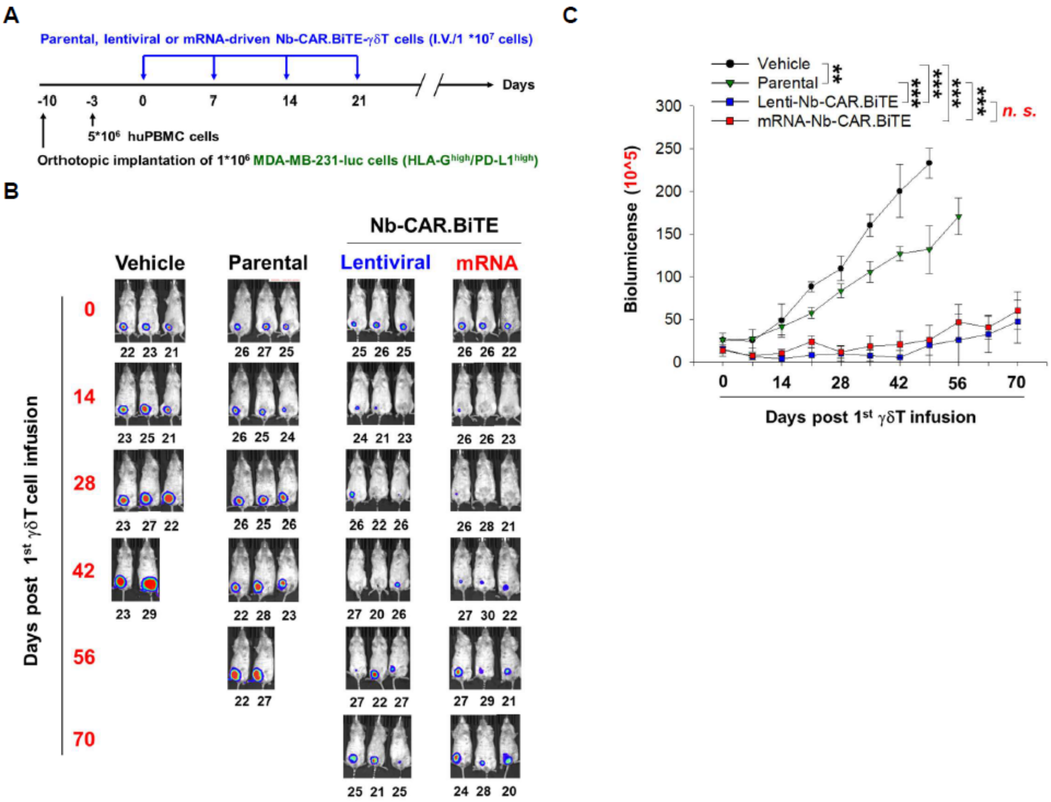

Supplementary Figure 5. Nb-CAR.BiTE-γδT cells are more effective than Nb-CAR-γδT cells combined with systemic PD-L1 blockade *in vivo*. A) Protocol for evaluating the anti-tumor activity of mRNA-driven Nb-CAR.BiTE-γδT cells and Nb-CAR-γδT cells combining with atezolizumab in PBMC-CDX-NSG mice bearing orthotopic HLA-G^{high}/PD-L1^{high} TNBC. Seven days after inoculation with 1 × 10^6 tumor cells, the mice received a tail vein injection of PBMCs (5 × 10^6 cells/mouse). Three days later, the parental (*n* = 5), Nb-CAR (*n* = 10), Nb-CAR.BiTE-γδT cells (1 × 10^7 cells/mouse) (*n* = 10), or Nb-CAR-γδT cells plus atezolizumab (1 × 10^7 cells and 20 mg/kg each mouse) (*n* = 10) were injected through tail vein once a week for 4

weeks. B, C) Superior anti-tumor activity of Nb-CAR.BiTE-γδT cells than Nb-CAR-γδT cells combining atezolizumab in the PBMC-CDX-NSG mouse model. The tumor growth of the orthotopically inoculated MDA-MB-231 were monitored weekly through IVIS. Data are shown as the mean ± SD; *p < 0.05; **p < 0.01; and ***p < 0.001 based on the Kaplan–Meier method and log-rank test.

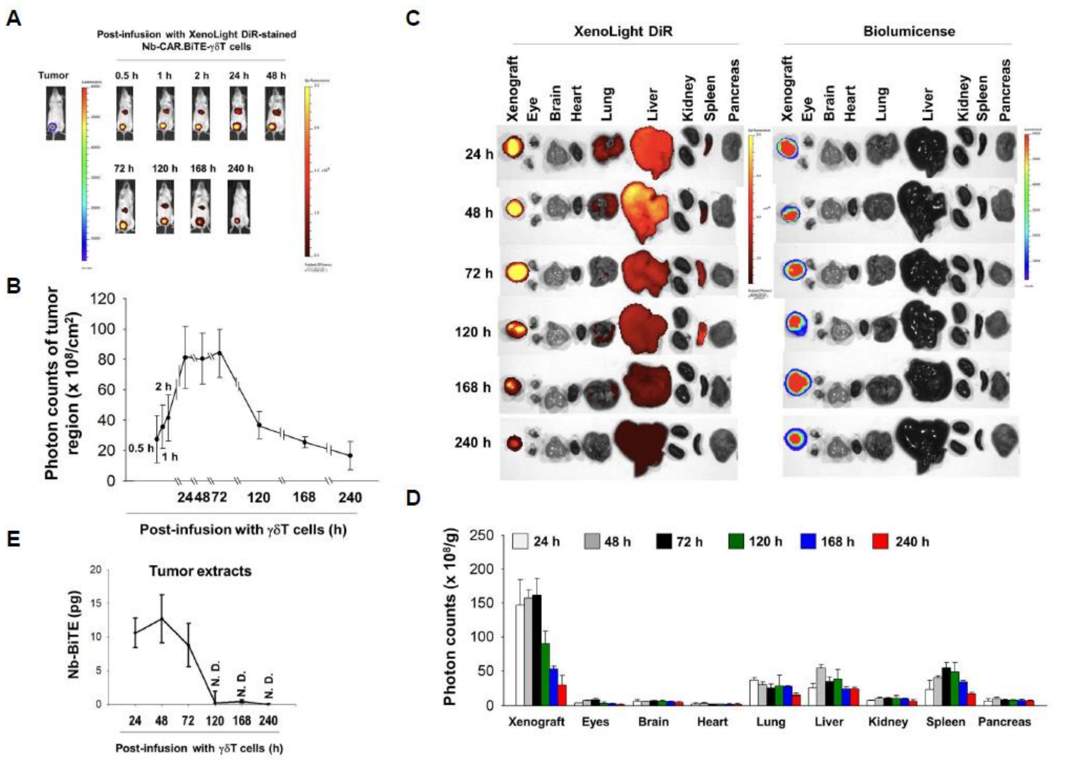

Supplementary Figure 6. Persistence of mRNA-engineered Nb-CAR.BiTE-γδT cells *in vivo*. A-D) Infused Nb-CAR.BiTE-γδT cells accumulated in the local breast tumor site and declined after 3 days. XenoLight™ DiR dye-stained Nb-CAR.BiTE-γδT cells (1 × 10^7 cells/mouse) were infused into MDA-MB-231 tumor-bearing NSG mice (n = 3 in each group) via tail vein injection. A,B) Fluorescent signals of each mouse were monitored by IVIS at the indicated time points, and B) fluorescent signals of tumor regions were quantified. C,D) Mice were sacrificed at the indicated time points, C) organs and tumor tissues were harvested to measure fluorescent signals by IVIS, and D) quantitative results were identified and

normalized per gram of tissue samples. E) Nb-BiTE was only detectable within 3 days after infusion of Nb-CAR.BiTE-γδT cells in PBMC-CDX-NSG mice bearing MDA-MB-231 tumors. The contents of PD-L1/CD3ε Nb-BiTE in the xenografted tumor tissue extracts (5 μg) were determined by an ELISA-based coating with PD-L1 recombinant protein. Data represent the mean ± SD, n = 3; results lower than 4 pg were considered non-detectable (N.D.).

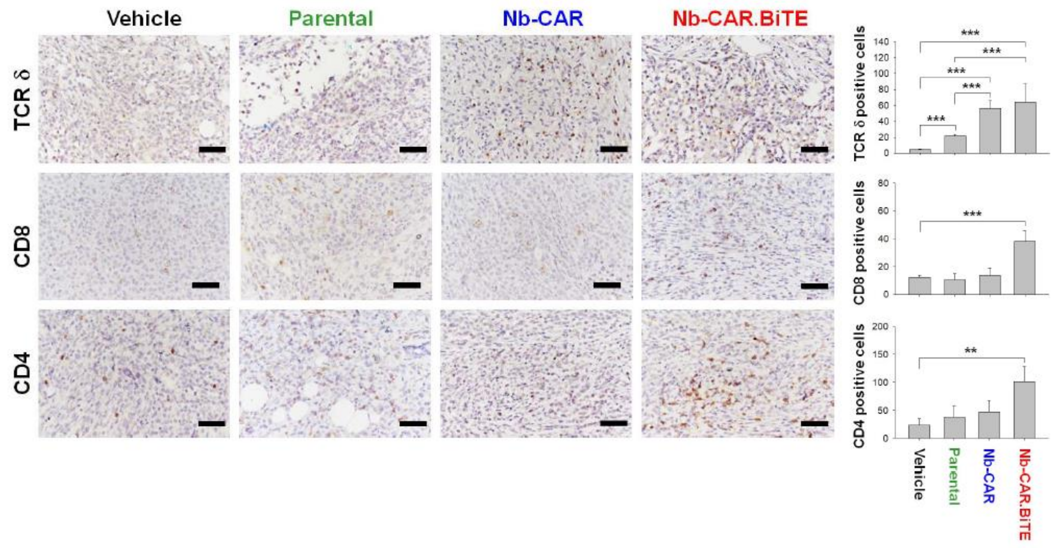

Supplementary Figure 7. mRNA-driven Nb-CAR.BiTE-γδT recruits bystander cells into tumor lesions. The PBMC-humanized NSG mouse model bearing orthotopic MDA-MB-231 tumors (n = 5) received tail vein injections weekly with or without parental, mRNA-engineered Nb-CAR or Nb-CAR.BiTE-γδT (1×10^7 cells in 100 μL PBS) for 4 weeks. Seven days after the last infusion, the tumors were harvested for detecting the infiltrated immune cells by IHC using specific antibodies against TCRδ, CD4 and CD8. The images were taken at 400 × HPF (left panel) and the positive cells were quantified (right panel). Results are representative of at least three independent experiments. Data represent the mean ± SD, n = 5; *p < 0.05; **p < 0.01; and ***p < 0.001 based on Student’s t-test.

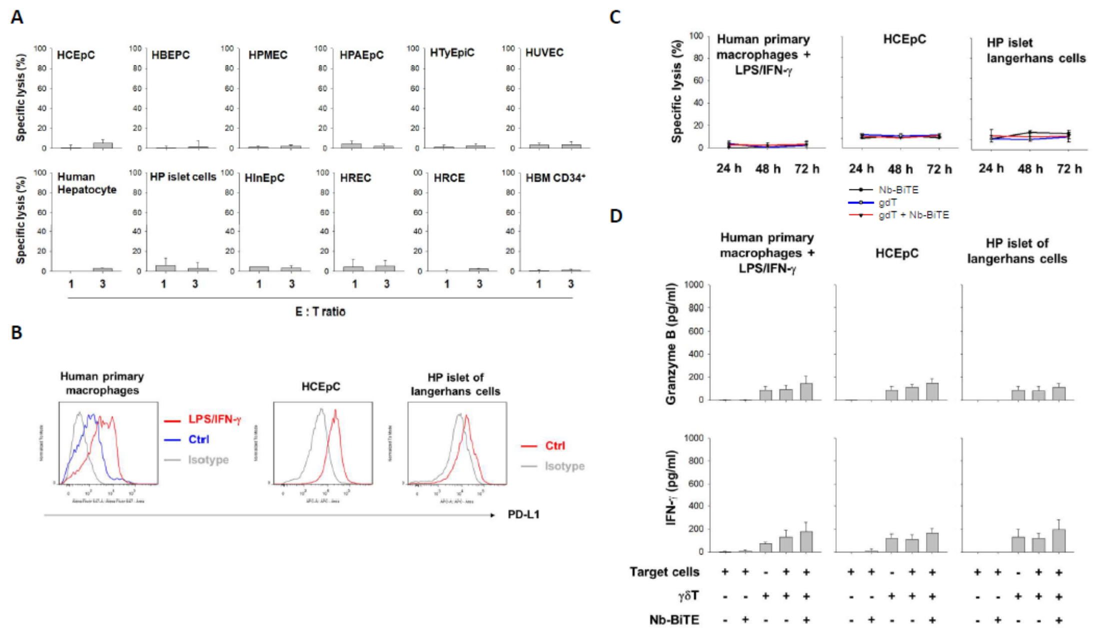

Supplementary Figure 8. mRNA-engineered Nb-CAR.BiTE-γδT cells and Nb-BiTE did not show obvious toxicity *in vitro.* A) Nb-CAR.BiTE IVT mRNA-electroporated γδT did not induce apparent cytotoxicity in various human primary cell cultures (2 donors of each tissue type). HUVEC, HRCE, HREC, HCEpC, HTyEpiC, HPAEpiC, HPMEC, human hepatocyte, HBEpC, HP islet cells, HInEpC, and HBM CD34$^+$ cells (1×10^5 cells for each) were individually cocultured with mRNA-driven Nb-CAR.BiTE-γδT at E:T ratios of 1:1 or 3:1 for 72 h. The cytotoxicity was then determined by LIVE/DEAD cell-mediated cytotoxicity assay. B) Expression levels of PD-L1 on macrophages pretreated 48 h with or without LPS (100 ng/ml)/IFN-γ (10 ng/ml), on HCEpC and HP islet of Langerhans cells were assessed by flow cytometry analysis. C,D) Primary macrophages, HCEpC, or HP islet of Langerhans cells were cocultured with or without the γδT cells at an E:T ratio of 3:1 in the presence or absence of recombinant Nb-BiTE (1 ng/ml). C) After 24, 48, or 72 h co-incubation, the induced cytotoxicity was determined by LIVE/DEAD cell-mediated cytotoxicity assay and analyzed via flow cytometry. D) Supernatants were harvested after 48 h to detect the levels of granzyme B and IFNγ by ELISA. Data are shown as the mean ±

SD, n = 4; paired Student’s t-test.

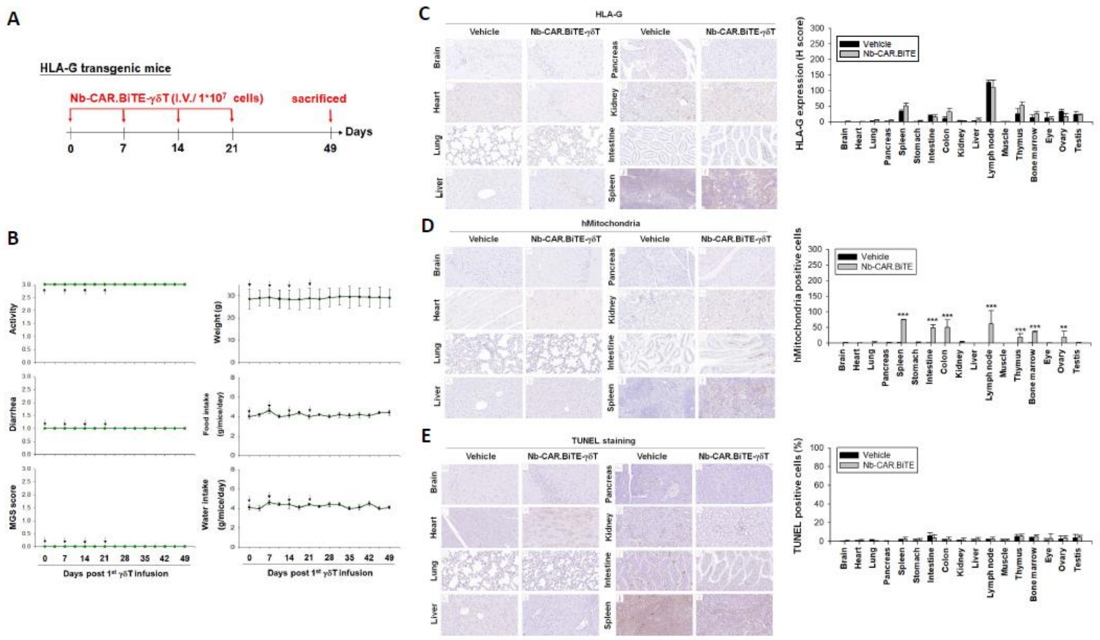

Supplementary Figure 9. mRNA-engineered Nb-CAR.BiTE-γδT cells have no obvious toxicity *in vivo*. A) Protocol for evaluating the toxicity induced by mRNA-driven Nb-CAR.BiTE-γδT infusion in HLA-G transgenic mice. HLA-G transgenic mice (*n* = 5) received tail vein injections with or without mRNA-driven Nb-CAR.BiTE-γδT cells (1×10^7) once a week for 4 weeks. At 28 days after the final injection, the mice were sacrificed and all the indicated organs and tissues were collected for subsequent analysis. B) Treatment with mRNA-driven Nb-CAR.BiTE-γδT cells did not increase the toxicity in HLA-G transgenic mice. The body weight, food intake, activity, presence of diarrhea, and MGS scores of the mRNA-driven Nb-CAR.BiTE-γδT-treated male and female HLA-G transgenic mice were assessed twice a week until 49 days after the first infusion and presented as individual plots. Food intake was measured as the daily average intake of each mouse. Arrows representative of the days of Nb-CAR.BiTE-γδT infusion. C-E) Repeated infusion with mRNA-driven Nb-CAR.BiTE-γδT did not cause obvious cell damage even in tissues indicating detectable level of HLA-G or accumulation of γδT cells. C)

HLA-G expression in each organ was detected by IHC staining. D) Presence of human cells was determined by IHC staining using a specific antibody against human mitochondria (hMitochondria). E) Apoptotic cells were detected by TUNEL assay, and positive apoptotic cells were identified based on brownish/black nuclear staining. We quantified the recorded H scores of HLA-G, the normalized TUNEL-positive cells to total cells, and the numbers of hMitochondria-positive cells in each HPF. (right panels, D-F). Results are representative of at least three independent experiments. Data represent the mean ± SD, n = 5; $**p < 0.01$ and $***p < 0.001$ based on paired Student's t-tests.

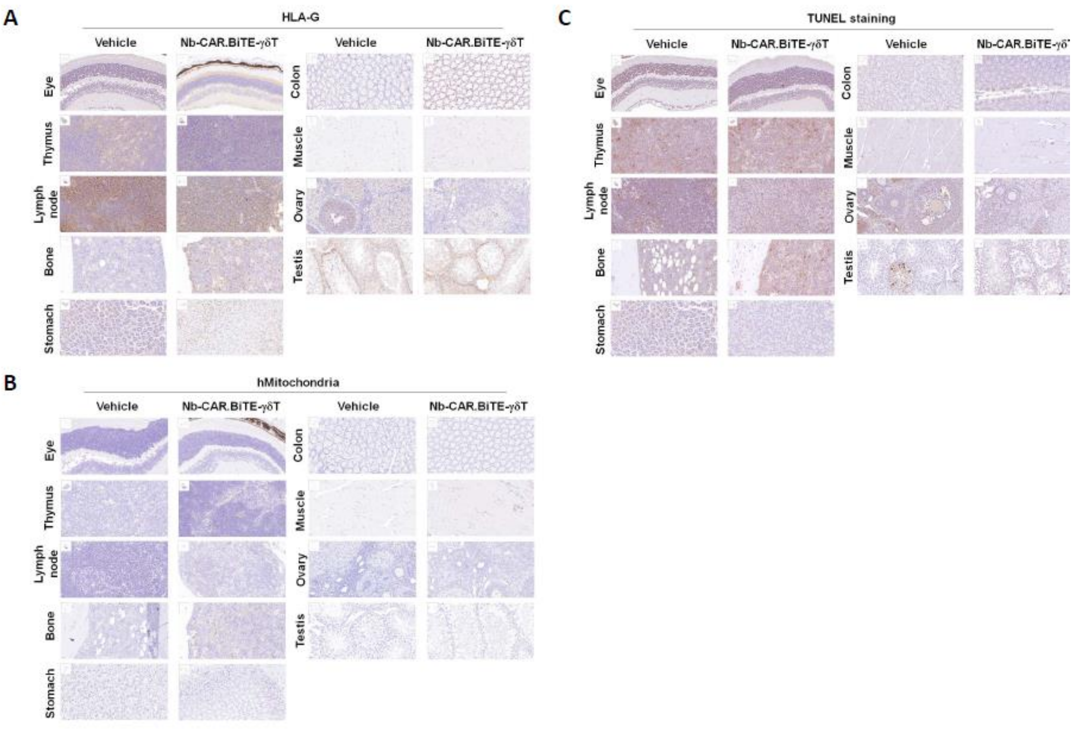

Supplementary Figure 10. mRNA-driven Nb-CAR.BiTE-γδT cells have no obvious histotoxicity *in vivo*. A-C) Repeated infusions with mRNA-driven Nb-CAR.BiTE-γδT did not cause tissue damage, even where HLA-G was detectable or where γδT cells accumulated. A) Expression of HLA-G in each organ was detected by IHC staining. B) Presence of human cells in each organ was determined by IHC staining using

hMitochondria specific antibodies. C) Apoptotic cells were determined by TUNEL staining. The quantified H scores of HLA-G, numbers of hMito-positive cells, and normalized TUNEL-positive cells to total cells in each HPF are shown in the right panels of **Supplementary Figure 9D-F.**
